# Liver Tumor Segmentation Based on Multi-Scale Deformable Feature Fusion and Global Context Awareness

**DOI:** 10.3390/biomimetics10090576

**Published:** 2025-09-01

**Authors:** Chenghao Zhang, Lingfei Wang, Chunyu Zhang, Yu Zhang, Jin Li, Peng Wang

**Affiliations:** 1 College of Intelligent Systems Science and Engineering, Harbin Engineering University, Harbin 150001, China; 910381219@hrbeu.edu.cn (C.Z.); wanglingfei@hrbeu.edu.cn (L.W.); 2 College of Computer and Control Engineering, Qiqihar University, Qiqihar 161006, China; 03813@qqhru.edu.cn; 3 Innovation Center of Yangtze River Delta, Zhejiang University, Jiaxing 314100, China; zhangyhrb04@gmail.com; 4 Artificial Intelligence Energy Research Institute, Northeast Petroleum University, Daqing 163318, China

**Keywords:** liver tumor segmentation, deformable large kernel attention, context extraction, dual cross attention

## Abstract

The highly heterogeneous and irregular morphology of liver tumors presents considerable challenges for automated segmentation. To better capture complex tumor structures, this study proposes a liver tumor segmentation framework based on multi-scale deformable feature fusion and global context modeling. The method incorporates three key innovations: (1) a Deformable Large Kernel Attention (D-LKA) mechanism in the encoder to enhance adaptability to irregular tumor features, combining a large receptive field with deformable sensitivity to precisely extract tumor boundaries; (2) a Context Extraction (CE) module in the bottleneck layer to strengthen global semantic modeling and compensate for limited capacity in capturing contextual dependencies; and (3) a Dual Cross Attention (DCA) mechanism to replace traditional skip connections, enabling deep cross-scale and cross-semantic feature fusion, thereby improving feature consistency and expressiveness during decoding. The proposed framework was trained and validated on a combined LiTS and MSD Task08 dataset and further evaluated on the independent 3D-IRCADb01 dataset. Experimental results show that it surpasses several state-of-the-art segmentation models in Intersection over Union (IoU) and other metrics, achieving superior segmentation accuracy and generalization performance. Feature visualizations at both encoding and decoding stages provide intuitive insights into the model’s internal processing of tumor recognition and boundary delineation, enhancing interpretability and clinical reliability. Overall, this approach presents a novel and practical solution for robust liver tumor segmentation, demonstrating strong potential for clinical application and real-world deployment.

## 1. Introduction

Liver tumors are common hepatic lesions and pose significant clinical challenges. Based on their biological behavior, they are classified as either benign or malignant, with the latter referred to as liver cancer. Liver cancer is characterized by high malignancy, rapid proliferation, and a strong propensity for distant metastasis. In recent years, the incidence of liver cancer has continued to rise, which makes it one of the most pressing global public health challenges [1,2]. However, the early clinical symptoms of liver cancer are often nonspecific, and the small size of early lesions makes them difficult to detect accurately using computed tomography (CT). As a result, most patients are diagnosed at intermediate or advanced stages, significantly increasing treatment complexity and leading to uncertain prognoses [3,4].

Against this backdrop, there is an urgent need to develop more efficient and accurate diagnostic and therapeutic strategies to improve treatment outcomes and patient survival. Surgical resection remains the primary clinical approach for treating liver cancer, particularly demonstrating favorable outcomes for early-stage lesions. However, in cases where tumors are located in anatomically complex regions or exhibit poorly defined boundaries, precise intraoperative localization of the lesion becomes more challenging, increasing the technical complexity of surgery and the risk of postoperative complications [5]. To address these challenges, medical image analysis techniques have played an increasingly critical role in the auxiliary diagnosis and therapeutic planning of liver tumors. Among these, liver tumor segmentation serves as a central component of intelligent image processing, aiming to accurately delineate tumor boundaries and their spatial distribution from CT images, which is essential for enabling personalized and precise treatment. Accurate lesion segmentation via automated or semi-automated approaches not only facilitates the formulation of appropriate preoperative resection plans but also provides real-time intraoperative guidance, thereby enhancing the safety and completeness of surgical procedures [6,7].

Although significant progress has been made in liver tumor segmentation based on CT images in recent years, numerous challenges persist in clinical practice. A major challenge stems from the significant morphological heterogeneity of liver tumors, which exhibit considerable variations in size, shape, and spatial location, along with notable inter-patient variability. These morphological variations often result in irregular tumor contours and indistinct boundaries, with limited contrast between tumors and surrounding healthy tissues, thereby significantly complicating accurate segmentation [8,9]. Furthermore, liver tumors undergo spatiotemporal progression during disease development, such as continuous growth or morphological changes. These dynamic patterns not only increase the complexity of the segmentation task but also place greater demands on model stability and generalization across different pathological stages [10]. Moreover, CT image acquisition is often affected by noise, patient motion artifacts, and complex anatomical structures, which degrade image quality, blur boundaries, and reduce tissue contrast, thereby exacerbating the difficulty of distinguishing tumors from adjacent tissues [11]. In addition, accurate tumor annotation critically relies on clinical expertise. However, the highly variable tumor morphology introduces subjective variability in interpretation, and the annotation process itself is time-consuming and labor-intensive, which hinders the construction of large-scale, high-quality labeled datasets and limits further improvement of segmentation model performance [12].

In the early stages, liver tumor image segmentation primarily relied on manual delineation by clinicians, a time-consuming process that was prone to operator-dependent variability, resulting in a heightened risk of misdiagnosis or missed lesions and overall low efficiency [13]. With the rapid advancement of medical imaging technologies, liver tumor segmentation methods have undergone a significant transition from traditional techniques to automated approaches. Traditional segmentation methods [14,15,16,17,18,19,20], such as active contour models, clustering analysis, edge detection, and level set methods, have shown some success under specific conditions. However, these approaches heavily depend on manually defined parameters and handcrafted features, making them inadequate for handling the complex and heterogeneous morphology of liver tumors. Moreover, liver tumor segmentation often requires the integration of multiple traditional techniques and relies on intricate preprocessing and postprocessing steps, resulting in cumbersome workflows that fall short of clinical demands for efficiency and robustness.

With the rapid development of deep learning, convolutional neural network (CNN)-based segmentation methods have become mainstream in research, significantly advancing automated liver tumor segmentation. Inspired by the hierarchical information processing mechanisms of the biological visual system, the receptive field design and local connectivity of convolutional neural networks mimic the way simple cells in the visual cortex extract local features, thereby providing a biomimetic foundation for image segmentation tasks. Although CNNs have achieved remarkable progress in liver tumor segmentation, the downsampling operations involved often lead to the loss of important features, thereby compromising segmentation accuracy. To address the issue of feature loss due to downsampling, Shelhamer E et al. [21] proposed the Fully Convolutional Network (FCN), which mitigates this loss through feature summation and performs pixel-wise classification to enable end-to-end segmentation. Building on this framework, Sun C et al. [22] employed the FCN architecture for liver tumor segmentation. Christ P F et al. [23] introduced a cascaded FCN architecture, where the first FCN extracts the liver region, which is then used as input to a second FCN for tumor segmentation. Zheng S et al. [24] further enhanced the FCN by integrating deformable modeling based on Non-negative Matrix Factorization (NMF), which was used to refine tumor boundary contours. However, due to FCN’s limitations in feature fusion, it struggles to preserve spatial details adequately, prompting further exploration of more advanced network architectures to improve segmentation performance.

To address the limitations of FCNs in preserving spatial information, Ronneberger O et al. [25] proposed the U-shaped network, U-Net, which employs a symmetric encoder–decoder architecture and skip connections to integrate local details with global contextual information. U-Net has since been widely adopted in medical image segmentation. This architectural design draws inspiration from the cooperative functioning of feedforward and feedback pathways in the biological nervous system. The symmetric encoder–decoder structure emulates the hierarchical processing of the visual pathway, while the skip connections mimic the shortcut connections in the brain, effectively preserving multi-scale spatial features. For liver tumor segmentation, various modifications have been made to the original U-Net to enhance its capability in representing complex lesion regions. Sahli H et al. [26] applied U-Net to liver tumor segmentation and compared its performance with SegNet, demonstrating U-Net’s superior ability to handle tumor regions. Ayalew Y A et al. [27] optimized the U-Net architecture by modifying the number of filters and network depth to improve segmentation performance in liver tumor regions. With the introduction of residual networks (ResNet) and densely connected networks (DenseNet), researchers began integrating these architectures with U-Net to enhance feature extraction and gradient propagation, thereby improving segmentation accuracy in medical imaging. Seo H et al. [28] proposed an improved U-Net with residual paths for liver tumor segmentation. This architecture incorporates residual paths with deconvolution and activation modules into skip connections and adds convolutional layers in the decoder to enhance fine-grained feature fusion. Sabir M W et al. [29] introduced the ResU-Net, which embeds residual blocks into the U-Net encoder to improve the modeling capacity for tumor-specific features. Li X et al. [30] developed a hybrid densely connected U-Net that combines 2D and 3D DenseUNet architectures to extract intra-slice and inter-slice tumor features, which are then integrated through a feature fusion module to produce the final segmentation output. Despite the strong performance of U-Net and its variants in improving segmentation accuracy, challenges remain in the flexible fusion of multi-scale features and the effective integration of deep semantic information.

To further address the limitations of the traditional U-Net in deep feature integration and cross-layer information flow, Zhou Z et al. [31] proposed the U-Net++ architecture. This architecture redesigns the skip connections to more flexibly fuse encoder features while alleviating training difficulties associated with increased network depth. This improvement is inspired by the cross-level interconnections of biological neural networks, akin to the dense lateral connections among different cortical regions that enable information integration and complementation, thereby enhancing the model’s capacity for multi-scale feature fusion. Based on this improvement, Wang J et al. [32] applied U-Net++ to liver tumor segmentation using a two-stage strategy: first segmenting the liver region and then reusing the resulting liver mask as input for precise tumor segmentation. Peng Q et al. [33] proposed FLAS-UNet++ for liver tumor segmentation, which builds upon U-Net++ by introducing Atrous Spatial Pyramid Pooling (ASPP) in the fourth and fifth encoding layers to enhance boundary learning. Additionally, Focal Loss was incorporated to focus the model on tumor edges and address class imbalance. Although U-Net++ improves feature fusion, it still faces challenges in capturing complex tumor characteristics, highlighting the need for more refined mechanisms to enhance the network’s sensitivity to critical regions.

To overcome the limitations in feature representation of existing networks, researchers have explored the integration of attention mechanisms into U-Net and its variants. By embedding attention modules into the encoder, decoder, and skip connections, these models aim to enhance responsiveness to tumor regions. This development aligns with the selective attention mechanisms of biological cognitive systems, emulating the neural regulation of the human visual system that enhances the processing of relevant information while suppressing irrelevant signals. Li H et al. [34] proposed a modified convolutional attention mechanism by incorporating attention modules into the skip connections, thereby improving sensitivity to liver tumor regions. Li J et al. [35] developed an Efficient Channel Attention-based Res-Unet++ for liver tumor segmentation, where the ECA-res module replaces conventional convolution layers in the encoder to enhance feature extraction and alleviate network degradation in deep architectures. Kushnure D T et al. [36] introduced HFRU-Net for liver tumor segmentation. This model reconfigures skip connections using local feature fusion mechanisms to enhance contextual information extraction. It incorporates a squeeze-and-excitation module within the skip connections and applies an Atrous Spatial Pyramid Pooling (ASPP) block at the bottleneck to strengthen deep spatial feature representation. Zang L et al. [37] applied a PCNN-based preprocessing step for image enhancement prior to liver tumor segmentation and integrated residual blocks with SE attention modules into both the encoder and decoder of U-Net to improve tumor-focused feature learning. Li W et al. [38] proposed SPA-Unet for liver tumor segmentation, embedding a channel attention-based spatial pyramid structure in the encoder to extract multi-scale features and capture contextual information. Additionally, a Residual Attention Block (RABlock) was incorporated into the decoder to accelerate convergence and emphasize key regions. Liu L et al. [39] proposed the SEU^2^-Net architecture, embedding the SE attention mechanism into the nested U-shaped structure of U^2^-Net to enhance the representation of liver tumor features. Although the aforementioned methods have demonstrated effectiveness in enhancing local feature representation, their reliance on convolutional operations limits their receptive fields, resulting in insufficient global context modeling. This highlights the need for architectures capable of capturing long-range dependencies.

To overcome the limitations of convolutional operations, researchers have begun exploring architectures with stronger global modeling capabilities. In this context, the Transformer architecture, with its self-attention mechanism, offers a natural advantage by directly capturing dependencies between arbitrary positions within an image, making it particularly effective for long-range context modeling. This has led to the emergence of hybrid architectures that integrate CNNs with Transformers. The design of such hybrid architectures is inspired by the brain’s multimodal information processing mechanisms. The CNN component emulates the visual cortex’s ability to extract local features, while the transformer’s self-attention mechanism mimics the establishment of global working memory and long-range dependencies between the prefrontal cortex and other brain regions, thereby enabling holistic understanding of complex visual scenes. Wang X et al. [40] proposed TransFusionNet for liver tumor segmentation. This model leverages a Transformer encoder to extract semantic features, while CNN and squeeze-and-excitation (SE) attention modules are used to capture local spatial and edge features. A multi-scale feature fusion module is employed to integrate these features and pass them to the decoder, thereby enhancing the network’s feature representation capacity. Li X et al. [41] proposed TransU^2^-Net, a liver tumor segmentation model based on a deeply nested U-shaped structure. Transformers were incorporated into the skip connections of U^2^-Net to enhance global context modeling, and a novel multi-scale fusion strategy was introduced to optimize the integration of features across different dimensions. Zhang C et al. [42] proposed SAA-Net for liver tumor segmentation, introducing a Scale-Axis Attention (SAA) mechanism as its core component. The SAA module enhances the sensitivity and expressiveness of multi-scale features through dynamic scale attention, while the axis attention module improves the efficiency of self-attention computation and strengthens convolutional attention, enabling the effective capture of long-range spatial dependencies.

With the continuous advancement of deep learning, the design paradigm of medical image segmentation networks has evolved from convolutional neural networks (CNNs) to Transformers, and more recently to Mamba-based architectures. Transformers, relying on the self-attention mechanism, demonstrate notable strengths in modeling global dependencies, but they suffer from high computational complexity. By contrast, Mamba, as an emerging state space model (SSM), achieves higher computational efficiency while more effectively modeling long-range sequence dependencies, showing greater adaptability in spatiotemporal feature representation. From a biomimetic perspective, the Mamba architecture draws inspiration from the brain’s dynamic modeling of continuous states during information processing, enabling efficient capture and integration of complex contextual information and providing new directions and technical support for medical image segmentation. Consequently, researchers have begun to explore hybrid CNN–Mamba frameworks to leverage their complementary strengths in local feature extraction and global dependency modeling. Jiang Y et al. [43] proposed MLLA-UNet for liver tumor segmentation, which incorporates a Mamba-inspired linear attention mechanism into the encoder to enhance feature extraction and employs a symmetric sampling structure to facilitate multi-scale feature fusion. Qamar S et al. [44] developed SAMA-UNet, which integrates adaptive Mamba-like aggregated attention into the encoder to strengthen feature representation, while employing a causal resonance learning mechanism to improve multi-scale feature integration. Zhu M et al. [45] introduced BPP-Net for liver tumor segmentation, inspired by the complex parallel and serial structures of the biological visual system. In this network, three Mamba modules of different scales are embedded in each encoding layer to extract multi-scale features, while a dual-channel fusion decoder progressively refines the segmentation results. These developments indicate that hybrid architectures have become a prevailing trend in medical image segmentation research, including CNN–Transformer [46], CNN–attention [47], and CNN–Mamba frameworks [48,49], offering important insights and technical references for liver tumor semantic segmentation. U-Net has been widely adopted and recognized as a baseline model in liver tumor semantic segmentation tasks. To further improve segmentation performance, researchers have continued to explore U-Net-based enhancements, primarily focusing on optimizing the encoder and skip connections to strengthen feature extraction and fusion capabilities. However, existing methods still suffer from several limitations: (1) Due to the highly heterogeneous and irregular morphology of liver tumors, the standard U-Net and its derivatives exhibit limited feature extraction capacity. Traditional network architectures struggle to capture the high variability of tumor boundaries and the morphological diversity of lesions, resulting in inadequate representation of complex tumor structures. (2) Most current improvements concentrate on localized refinements of skip connections, while overlooking the complementary relationships among multi-scale features within the encoder. This oversight leads to significant semantic inconsistency between the encoder and decoder, which severely hinders effective feature transmission. (3) U-Net and its variants are mostly based on CNN architectures, where the inherent locality of convolution operations limits the network’s global receptive field. This local constraint significantly hampers the model’s ability to perceive the overall tumor structure, thus impeding accurate semantic segmentation.

To address the limitations in liver tumor semantic segmentation, this study proposes enhancements to the U-Net architecture from three perspectives: feature extraction, feature fusion, and global perception. The specific improvements are as follows: (1) A Deformable Large Kernel Attention (D-LKA) mechanism is introduced in the encoder stage, which dynamically adjusts the geometric deformation and receptive field distribution of the convolutional kernels to enable adaptive focusing on tumor regions. This design enhances the model’s ability to extract features from tumors with ambiguous boundaries and improves its sensitivity to small-scale lesions. (2) A Dual Cross Attention mechanism (DCA) is employed to replace traditional skip connections, facilitating the modeling of long-range dependencies and strengthening global contextual representation. This design enables deep integration across encoder feature hierarchies and promotes the fusion of high-level semantic information with low-level spatial details, thereby alleviating semantic gaps during feature transmission. (3) A multi-scale Context Extraction (CE) module is innovatively integrated into the bottleneck layer. Building upon the global modeling capacity provided by the Dual Cross Attention mechanism, this design further enhances the model’s ability to capture multi-scale contextual information and improves its overall perception of global features.

It is important to note that although the D-LKA, CE, and DCA mechanisms have each been proven effective in their original contexts, the innovation of this study lies in their tailored integration, which achieves a synergistic effect. Within the encoder, D-LKA enables dynamic adaptation to tumor morphological heterogeneity, focusing on critical regions and mitigating boundary ambiguity. The CE module enhances tumor-related channel semantics during deep feature extraction while suppressing noise. DCA restructures the information flow between the encoder and decoder, not only replacing conventional skip connections but also establishing bidirectional long-range dependencies across levels, thereby enabling deep integration of locally adaptive features, channel-level semantic enhancement, and multi-scale global interactions. This synergistic design substantially enhances the model’s overall ability to represent complex liver tumor structures, surpassing the performance achievable by individual modules or simple stacking, and serves as the key driver of the proposed method’s performance improvement.

## 2. Methods

### 2.1. Baseline Network: U-Net

**Figure 1:**
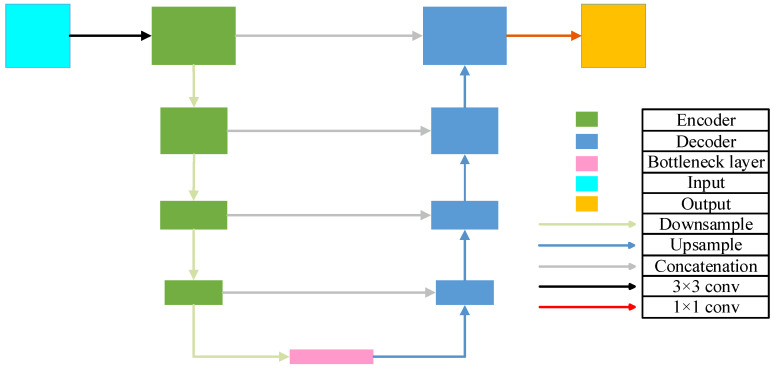
The architecture of U-Net.

U-Net [25] is a widely used convolutional neural network architecture for image segmentation, characterized by its symmetric U-shaped design. This structure integrates an encoder (downsampling path), a bottleneck, and a decoder (upsampling path), and connects feature maps from corresponding encoder and decoder layers via skip connections, as illustrated in Figure 1.

The encoder consists of multiple convolutional blocks, each typically comprising a convolutional layer, an activation function, and a pooling layer. Let $E_{i}$ denote the output feature map of the block i in the encoder. The encoder output is expressed as shown in Equation (1):

(1) $$E_{i}=Pool(\mathrm{ReLU}(Conv(E_{i-1})))$$

where Conv denotes the convolution operation, ReLU is the activation function, Pool represents the pooling operation, and $E_{0}$ is the input image.

The decoder also consists of multiple convolutional blocks, each typically comprising an upsampling layer (a transposed convolution followed by a convolution), a convolutional layer, an activation function, and a skip connection from the corresponding encoder layer. Let $D_{i}$ denote the output feature map of the block i in the decoder. The decoder output is defined as shown in Equation (2):

(2) $$D_{i}=\mathrm{ReLU}(Conv(UpSample(D_{i-1})©Crop(E_{n-i+1})))$$

where UpSample denotes the upsampling operation, © represents feature map concatenation, and Crop refers to the necessary cropping operation to ensure spatial dimension alignment. n is the number of blocks in the encoder, and $D_{0}$ is the initial decoder feature map, typically obtained by upsampling from $E_{n}$, the output of the final encoder block, i.e., the bottleneck layer.

The final block of the decoder is typically followed by one or more convolutional layers to generate the final segmentation map. Let S denote the output segmentation map; the final segmentation result is expressed as shown in Equation (3):

(3) $$S=\mathrm{Softmax}(Conv(D_{n}))$$

where Softmax is used to convert the output into a probability map, where the sum of probabilities across all classes for each pixel equals 1.

### 2.2. Overall Network Architecture with Multi-Scale Deformable Feature Fusion and Global Perception

**Figure 2:**
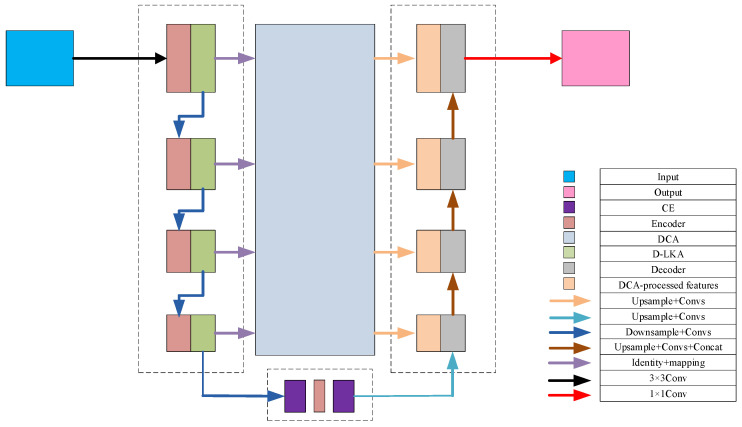
Overall network architecture with multi-scale deformable feature fusion and global perception.

Based on the U-Net architecture, this study proposes an enhanced segmentation network, D1CD2U-Net, by integrating a Deformable Large Kernel Attention mechanism (D1), a Context Extraction module (C), and a Dual Cross Attention mechanism (D2). The composite structure of D1CD2U-Net provides strong feature modeling capabilities. To facilitate an intuitive understanding of the proposed feature processing flow, the overall architecture of the improved network is illustrated in Figure 2.

To address the task of liver tumor segmentation, this study proposes a novel segmentation network framework that integrates multi-scale deformable features with global perception capabilities. Given the complex and heterogeneous morphology of liver tumors, the model incorporates a Deformable Large Kernel Attention mechanism, which adaptively adjusts the receptive field size and dynamically focuses on key tumor regions. This enhances the model’s ability to represent heterogeneous tumor shapes, improving both its adaptability and robustness. The mechanism thus provides more discriminative features for accurate subsequent segmentation.

Next, a Context Extraction module is introduced at the bottleneck layer of the encoder to enhance the capture of global contextual information. This enables the model to acquire a more comprehensive and in-depth understanding of both the liver and its tumor environment, laying a solid foundation for subsequent feature fusion and image reconstruction.

To further optimize the feature transmission path, a Dual Cross Attention mechanism is employed to replace traditional skip connections. This mechanism operates on two levels: (1) Channel Self-Attention, which emphasizes interactions among different feature channels, enhancing salient features while suppressing irrelevant noise; and (2) Spatial Self-Attention, which captures interdependencies across different spatial locations to facilitate effective spatial information exchange. By passing encoder features through this Dual Cross Attention mechanism, the network achieves deep integration and fusion across spatial and channel dimensions, significantly enriching feature representation.

Finally, the features processed by the dual attention mechanism are organically fused with the global information extracted from the bottleneck layer, resulting in a more comprehensive and semantically rich feature representation. These features are then fed into the decoder, providing a strong foundation for the subsequent image reconstruction process. During decoding, the tumor’s fine structures are gradually restored through layer-wise upsampling and feature fusion, ultimately achieving accurate image segmentation. The entire framework, through these carefully designed components, enables efficient and precise processing of liver tumor images. The feature transmission process of the model is formulated as shown in Equation (4):

(4) $$\begin{array}{l} input=F\in R^{C\times H\times W}\\ F_{E_{n}}=D-LKA(ConvF_{E_{n-1}})\\ F_{E_{5}}=CE(ConvF_{E_{4}})\\ F_{D_{n}}=DCAF_{E_{n}}©ConvF_{D_{n+1}}\\ output=Sigmoid(F_{D_{1}}) \end{array}$$

where $F_{E_{n}}$ denotes the encoder and bottleneck layers: for *n* = 1 to 4, it refers to the encoder layers, and for *n* = 5, it represents the bottleneck layer. $F_{D_{n}}$ denotes the decoder layers; CE represents the Context Extraction module; D−LKA stands for the Deformable Large Kernel Attention mechanism; and DCA denotes the Dual Cross Attention mechanism.

The three core modules proposed in this study are inspired by biological mechanisms, reflecting a biomimetic design philosophy. (1) The D-LKA module, designed for the encoding stage, is inspired by the selective attention mechanism of the visual nervous system. It emulates the ability of cortical neurons to dynamically adjust the size and shape of receptive fields in response to stimuli. By introducing geometric deformations to convolutional kernels, it achieves adaptive receptive field distribution, analogous to the visual system’s enhanced focus on specific targets. This substantially improves the model’s ability to capture features of heterogeneous tumor structures and blurred boundaries. (2) The DCA module replaces conventional skip connections and is inspired by long-range connections and global working memory mechanisms in neural networks of the brain. By leveraging self-attention, DCA dynamically establishes long-range dependencies between features, simulating the prefrontal cortex’s self-organizing process of global integration and selection. Through bidirectional pathways between the encoder and decoder, it effectively mitigates semantic inconsistencies and promotes deep fusion of multi-level features, resembling the active coordination and modulation of perceptual information in advanced cognitive systems. (3) The CE module, integrated into the bottleneck layer, draws inspiration from the multi-scale parallel processing strategy of the biological visual system. It mimics the visual system’s ability to simultaneously process high-resolution information from the fovea and broad contextual cues from peripheral vision. By fusing multi-scale features, it enhances the model’s capacity to capture semantic dependencies across different ranges, thereby improving its overall perception and discrimination of complex lesion regions.

The core challenges of liver tumor CT segmentation can be summarized as follows: (1) high morphological heterogeneity and blurred lesion boundaries; (2) the dynamic spatiotemporal evolution of lesions; (3) the susceptibility of CT image quality to interference; and (4) the scarcity and subjectivity of high-quality annotated data. To address these challenges, this study proposes an improved framework: the D-LKA module adaptively responds to morphological variations in lesions, enabling precise focus on blurred boundaries. A multi-scale CE mechanism captures semantic information across different scales, thereby enhancing the model’s adaptability and robustness to lesion evolution. DCA strengthens global context modeling, mitigating the adverse effects of image quality variations.

### 2.3. Feature Extraction Architecture Design

Liver tumors often exhibit pronounced deformable characteristics, imposing stringent demands on the accuracy and robustness of segmentation models. The U-Net architecture, a classic model in medical image segmentation, demonstrates strong feature extraction capabilities due to its distinctive encoder–decoder framework. However, in the challenging task of liver tumor segmentation, the fixed receptive field convolution strategy employed by the traditional U-Net often fails to adequately capture subtle variations along tumor boundaries and the complex interactions between tumors and surrounding tissues, thereby limiting further improvements in segmentation performance.

To overcome this bottleneck, recent research trends have focused on integrating attention mechanisms into the U-Net architecture [35,38,40,42]. Inspired by the human visual system’s information processing, attention mechanisms dynamically adjust the weighting of input data, enabling the model to intelligently focus on the most critical regions within an image. Common attention mechanisms include channel attention [50], spatial attention [51], and self-attention [52], each enhancing the model’s representation capability from different perspectives. Channel attention improves performance by optimizing the contribution of each feature map channel; spatial attention emphasizes the saliency of each spatial location within the feature map; and self-attention captures global information by computing correlations between any two points in the feature map. However, given the irregular shapes and blurred boundaries of liver tumors, these conventional attention mechanisms still have certain limitations.

**Figure 3:**
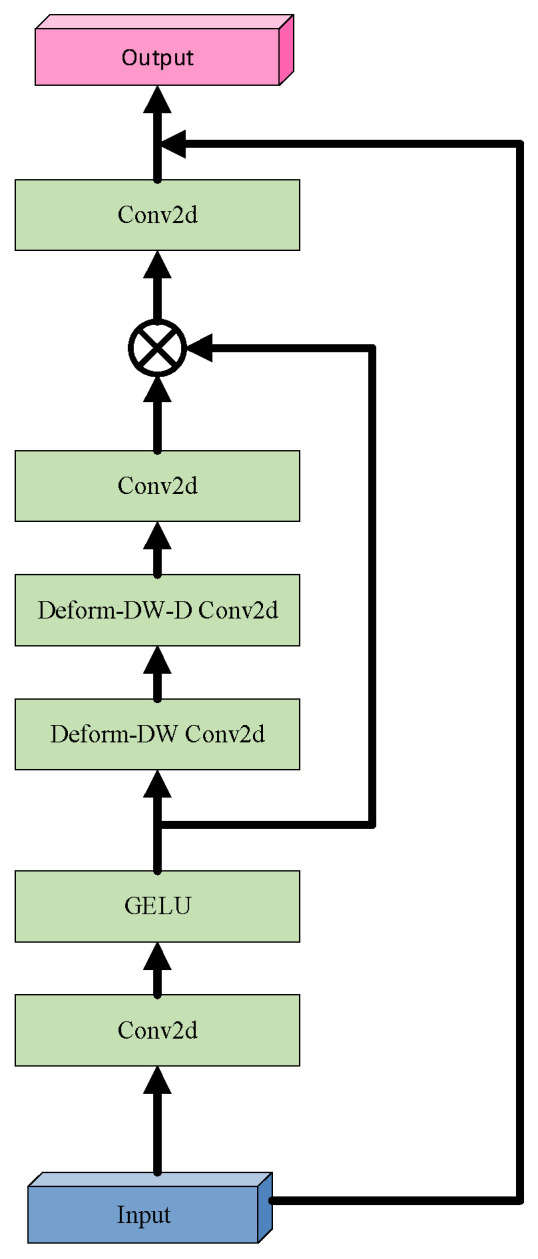
Architecture of the Deformable Large Kernel Attention (D-LKA) module.

In response, this study introduces the Deformable Large Kernel Attention (D-LKA) mechanism, integrated into the encoder layers of U-Net. The core strength of D-LKA lies in its unique architectural design and powerful information aggregation capabilities [53]. By incorporating deformable convolution techniques, D-LKA flexibly adjusts the shape and size of convolution kernels to more precisely capture subtle variations in tumor regions and their surrounding environment. Additionally, its large kernel design ensures the model captures richer contextual information, which is critical for distinguishing tumors from normal tissue. Moreover, D-LKA preserves fine local feature details, helping maintain clear and continuous segmentation boundaries, thereby further enhancing segmentation quality. The structural design of D-LKA is illustrated in Figure 3.

Before delving into D-LKA, it is essential to revisit the Large Kernel Attention (LKA) mechanism. LKA simulates the receptive field of self-attention by decomposing a large convolutional kernel and incorporates an attention mechanism to enhance the focus on target features. Specifically, a convolution with kernel size *K* is decomposed into a combination of three convolutions: a depthwise convolution with kernel size Kd, a depthwise dilated convolution with kernel size (2d−1) and dilation rate d, and a channel-wise convolution (1×1 convolution). Firstly, LKA employs a small depthwise convolution to capture local image features. Secondly, it applies a depthwise dilated convolution to expand the receptive field, enabling the capture of broader contextual information without increasing the number of parameters. Finally, a 1×1 convolution is used to integrate the features and produce the final attention map, facilitating cross-channel information fusion and feature aggregation.

D-LKA innovatively integrates the core concept of deformable convolution into the LKA framework by introducing a dynamic sampling grid, enabling flexible deformation of the convolutional kernel. Specifically, an auxiliary convolutional layer learns spatial offset fields from the input features autonomously, generating geometrically adaptive kernels. This feature-driven approach achieves two key properties: (1) sampling point locations undergo continuous spatial transformations based on the target morphology; (2) the kernel shape dynamically adapts to local structural features. Technically, the offset prediction network shares the same kernel size and dilation rate as its corresponding convolutional layer, ensuring scale consistency between geometric deformation and semantic features. For sampling points with non-integer coordinates, bilinear interpolation is employed for sub-pixel feature extraction, ensuring continuity and differentiability during deformation. This innovative design enables D-LKA to simultaneously achieve three critical functions: precise local detail perception, effective global context integration, and adaptive modeling of target shapes, thereby significantly enhancing the model’s capability to represent complex liver tumor boundaries. The mathematical formulation of D-LKA is presented in Equation (5):

(5) $$\begin{array}{l} input=F\in R^{C\times H\times W}\\ F_{1}=Conv_{1\times 1}(F)\\ F_{2}=GELU\left(F_{1}\right)\\ F_{3}=DDW\;-\;Conv(F_{2})\\ F_{4}=DDW\;-\;D\;-\;Conv(F_{3})\\ Attention=Conv_{1\times 1}(F_{4})\\ F_{5}=Attention⊗F_{2}\\ output=conv_{1\times 1}F_{5}+F \end{array}$$

where $F\in R^{C\times H\times W}$ denotes the input feature; *GELU*(*x*) represents the *GELU* activation function; $Couv_{1\times 1}$ refers to 1×1 a 2D convolution;DDW - Couv indicates a deformable depthwise convolution; DDW - D - Couv denotes a deformable depthwise dilated convolution; and $Attention\in R^{C\times H\times W}$ corresponds to the attention map, where each value reflects the relative importance of the associated feature. The operator ⊗ represents element-wise multiplication.

### 2.4. Design of the Feature Fusion Architecture

In the U-Net architecture, skip connections play a crucial role in facilitating information flow between the encoder and decoder, enabling effective fusion of features across different levels. Existing improvements primarily focus on integrating various types of attention mechanisms or multi-scale representations within the skip connections. However, these approaches still fall short in fully bridging the semantic gap between encoder and decoder features [54,55]. This semantic gap often results in over-segmentation or under-segmentation in liver tumor delineation, leading to false positives or false negatives. Moreover, the convolutional architecture of U-Net is inherently limited to local feature extraction, which constrains its ability to model global context—an essential aspect for fine-grained segmentation tasks. Addressing these issues requires the design of more sophisticated and effective feature fusion mechanisms to enhance the model’s ability to capture broad contextual information, thereby mitigating the semantic gap and improving global modeling capabilities.

**Figure 4:**
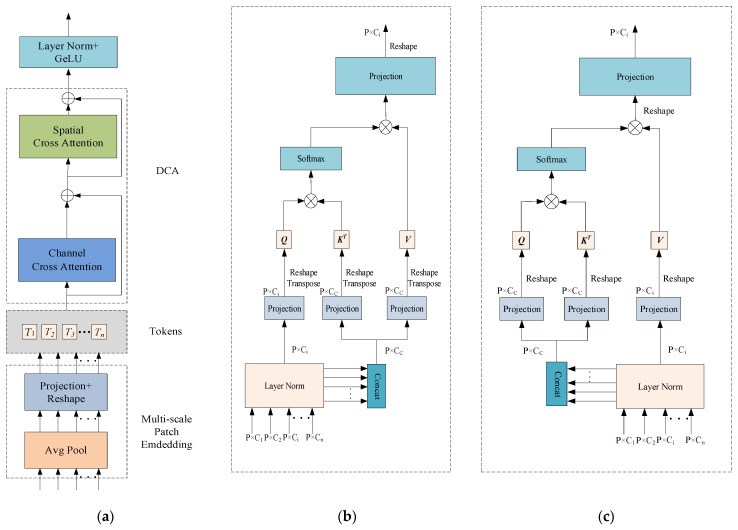
The Dual Cross Attention (DCA) mechanism; (**a**) overall architecture of the Dual Cross Attention (DCA) mechanism; (**b**) Channel Cross Attention (CCA) module; (**c**) Spatial Cross Attention (SCA) mechanism.

The Dual Cross Attention (DCA) mechanism Is an attention module designed to integrate multi-scale encoder features by capturing global dependencies across both channel and spatial dimensions [56]. Specifically, the Channel Cross Attention (CCA) module first performs cross-channel attention operations among channel tokens to capture global channel-wise dependencies. Then, the Spatial Cross Attention (SCA) module captures spatial dependencies via Cross Attention among spatial tokens, enhancing the model’s sensitivity to fine-grained spatial structures. These refined encoder features are subsequently upsampled and fused with the corresponding decoder features, enabling effective multi-level feature integration. By establishing cross-scale and cross-channel associations, this mechanism effectively integrates spatial and channel information, alleviating the feature mismatch caused by the semantic gap in traditional skip connections and improving segmentation performance. Based on these advantages, this study replaces traditional skip connections with the DCA mechanism to enhance the model’s perception of global structures. The detailed structure is illustrated in Figure 4.

The architecture of the Dual Cross Attention (DCA) mechanism remains consistent with the number of encoder stages. In other words, given *n* + 1 multi-scale encoder stages, the DCA module takes the multi-scale features from the first *n* stages as input to enhance feature representation and connects them to the corresponding *n* decoder stages. As illustrated in Figure 4, the DCA mechanism consists of two main stages. The first stage comprises a multi-scale embedding module that extracts encoder tokens from the input features. In the second stage, Channel Cross Attention and Spatial Cross Attention mechanisms are applied to these encoder tokens to capture long-range dependencies. Finally, the tokens are upsampled through a sequence of normalization and GeLU activation, and then fused with the corresponding decoder stages to enable effective cross-level feature integration.

#### 2.4.1. Patch Embedding Based on a Multi-Scale Encoder

Patches are extracted from *n* multi-scale encoder stages. Given *n* encoder stages at different scales, the input size is $E_{i}\in R^{C^{i}\times \frac{H}{2^{i-1}}\times \frac{H}{2^{i-1}}}$, and the patch size is $P_{i}^{S}=\frac{P^{S}}{2^{i-1}}$, where i=1,2,⋅⋅⋅,n corresponds to the scale levels. Average pooling with a kernel size and stride of $P_{i}^{S}$ is applied to extract patches. Subsequently, 1×1 depthwise separable convolution is employed on the flattened 2D patches to perform feature mapping. The resulting feature representation of the multi-scale embedding module is formulated as Equation (6):

(6) Ti=DConv1DEi(Reshape(AvgPool2DEi(DEi)))

where $T_{i}\in R^{P\times C_{i}}(i=1,2,\cdot \cdot \cdot ,n)$ denotes the flattened patches from the *i* encoder stage. Note that P represents the number of patches, which is the same across each $T_{i}$. This uniformity allows the application of Cross Attention mechanisms among these tokens.

#### 2.4.2. Channel Cross Attention

Each token $T_{i}(i=1,2,\cdot \cdot \cdot ,n)$ is processed using Channel Cross Attention(CCA). Specifically, each $T_{i}$ is first normalized via Layer Normalization (LN), then concatenated along the channel dimension to form $T_{c}$, which is used to generate the key and value, while $T_{i}$ itself serves as the query. Depthwise separable convolution is integrated into the self-attention mechanism to better capture local context while reducing computational complexity. To achieve this, all linear projections are replaced with 1×1 depthwise convolutional projections. The expressions for the queries, keys, and values in the Channel Cross Attention module are defined in Equation (7):

(7) $$\begin{array}{l} Q_{i}=DConv1D_{Q_{i}}(T_{i})\\ K=DConv1D_{K}(T_{C})\\ V=DConv1D_{V}(T_{C}) \end{array}$$

where $Q_{i}\in R^{P\times C_{i}},K\in R^{P\times Cc},V\in R^{P\times Cc}$ represent the mapped queries, keys, and values, respectively. Accordingly, the formulation of the Channel Cross Attention is provided in Equation (8):

(8) $$CCA(Q_{i},K,V)=Soft\max(\frac{Q_{i}^{T}K}{\sqrt{C_{C}}})V^{T}$$

where 1CC is a scaling factor designed to control the variance of the dot-product results, thereby mitigating the risk of gradient vanishing. The output of the Cross Attention mechanism is a weighted sum of the values, where the weights are determined by the similarity between the queries and the keys. Finally, the output of the Cross Attention is refined using depthwise separable convolution and then fed into the Spatial Cross Attention module.

#### 2.4.3. Spatial Cross Attention

Given the output $\overset{-}{T_{i}}\in R^{P\times C_{i}}(i=1,2,\cdot \cdot \cdot ,n)$ from the CCA module, layer normalization is applied followed by concatenation along the channel dimension. Unlike the CCA module, the concatenated token $\overset{-}{T_{C}}$ is used as both the query and key, while each $\overset{-}{T_{i}}$ serves as the value. A 1 × 1 depthwise separable convolution is applied to project the queries, keys, and values. The expressions for the queries, keys, and values in the Spatial Cross Attention (SCA) module are defined in Equation (9):

(9) $$\begin{array}{l} Q=DConv1D_{Q}(\overset{-}{T_{C}})\\ K=DConv1D_{K}(\overset{-}{T_{C}})\\ V_{i}=DConv1D_{V_{i}}(\overset{-}{T_{i}}) \end{array}$$

where $Q\in R^{P\times Cc},K\in R^{P\times Cc},V_{i}\in R^{P\times C_{i}}$ represent the mapped queries, keys and values, respectively. The formulation of the Spatial Cross Attention is presented in Equation (10):

(10) $$SCA(Q,K,V_{i})=Soft\max(\frac{QK^{T}}{\sqrt{d_{k}}})V_{i}$$

where 1dk is a scaling factor. In the multi-head setting, dk=CChC, where $h_{C}$ denotes the number of heads. The output of the *SCA* is refined using depthwise separable convolution to produce the final feature representation of the DCA mechanism. After layer normalization and GeLU activation, the *n* output tokens of the module are upsampled and connected to the corresponding layers in the decoder.

This study introduces a dual attention mechanism that differs from the traditional self-attention approach. Instead of generating attention maps separately at each stage, the DCA mechanism integrates multi-scale features from different encoder stages. This enables the model to capture long-range dependencies across various encoder levels, thereby narrowing the semantic gap between features extracted at different encoder layers, and ultimately mitigating the semantic inconsistency between the encoder and decoder.

### 2.5. Design of the Bottleneck Structure

Previous studies have primarily focused on optimizing the encoder, decoder, and skip connections to improve network performance [36,37,38], while the bottleneck layer—serving as a critical bridge between them—has often been overlooked. As a vital component of the U-Net architecture, the bottleneck layer not only encapsulates the most semantically rich features but also plays a pivotal role in effectively transmitting these features from the encoder to the decoder, which is crucial for ensuring segmentation accuracy. However, the original bottleneck design relies on conventional convolutions, which are inherently limited by a narrow receptive field. This restricts the network’s capacity to capture global contextual information, potentially compromising the comprehensiveness and precision of the segmentation results. Therefore, enhancing the bottleneck structure to better capture global context has become an important direction for improving the segmentation performance of U-Net. In this study, a Context Extraction (CE) mechanism is introduced into the bottleneck layer. This mechanism comprises two core modules: a Dense Atrous Convolution (DAC) module and a Residual Multi-kernel Pooling (RMP) module [57]. This design enables the efficient extraction of high-level semantic information, thereby enhancing the model’s contextual understanding.

**Figure 5:**
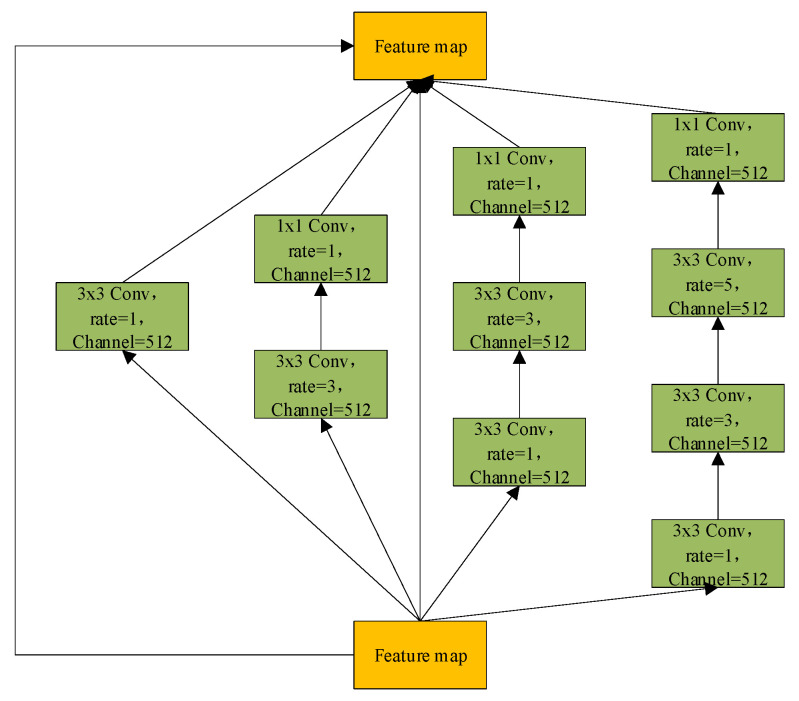
Dense Atrous Convolution (DAC) module.

The DAC (Dense Atrous Convolution) module combines atrous convolution, the Inception architecture, and the residual learning mechanism from ResNet. It is designed to enhance the model’s ability to capture multi-scale information while preserving feature integrity. The structure of the DAC module is illustrated in Figure 5. Specifically, the DAC module consists of four parallel convolutional branches, each employing a different dilation rate (rate = 1, 3, 1 + 3, and 1 + 3 + 5) to simulate multi-scale receptive fields ranging from local detail to global context. This design is inspired by the multi-branch parallelism of the Inception architecture, aiming to improve adaptability to complex structural variations through architectural diversity. In each branch, a preceding standard convolution is applied to reduce the computational burden of directly using large receptive-field atrous convolutions, while also enhancing feature diversity through linear projection. The multi-scale features extracted by each branch are then fused with the input feature map via element-wise addition across channels. This process draws on the residual connection mechanism from ResNet, which facilitates gradient propagation and mitigates information degradation in deep networks. In liver tumor segmentation tasks, where tumors often exhibit highly complex and irregular morphologies, the DAC module provides a robust feature foundation by leveraging large-receptive-field branches to capture contextual semantics and small-receptive-field branches to retain sensitivity to fine details. The mathematical formulation of the DAC module is presented in Equation (11):

(11) $$\begin{array}{l} input_{DAC}=x\in R^{C\times H\times W}\\ Y_{1}=Conv_{3\times 3}d1(x)\\ Y_{2}=Conv_{1\times 1}d1(Conv_{3\times 3}d3(x))\\ Y_{3}=Conv_{1\times 1}d1(Conv_{3\times 3}d3(Conv_{3\times 3}d1(x)))\\ Y_{4}=Conv_{1\times 1}d1(Conv_{3\times 3}d5(Conv_{3\times 3}d3(Conv_{3\times 3}d1(x))))\\ Y_{5}=x\\ output_{DAC}=Y_{1}+Y_{2}+Y_{3}+Y_{4}+Y_{5} \end{array}$$

where $Conv_{3\times 3}$ denotes a 3 × 3 convolution, $Conv_{1\times 1}$ represents a 1 × 1 convolution, and $d_{n}$ indicates the dilation rates used in different branches.

**Figure 6:**
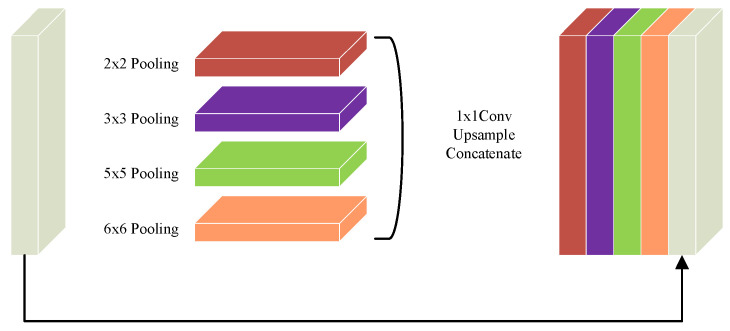
Residual Multi-kernel Pooling (RMP) module.

To address the high computational cost associated with the enhanced feature representation in the DAC module, this study introduces a Residual Multi-kernel Pooling (RMP) module as an optimization strategy. The structure of the RMP module is illustrated in Figure 6. The RMP module employs four different pooling kernels with sizes ranging from 2 × 2 to 6 × 6 to capture multi-scale receptive fields. This design enhances the model’s feature extraction capacity while reducing redundant computations and the number of parameters. Subsequently, a 1 × 1 convolution is applied to perform dimensionality reduction and feature refinement. An upsampling operation then aligns all feature maps to a unified spatial resolution to ensure spatial consistency. During the fusion stage, the multi-scale features are concatenated with the original feature map, enabling efficient integration of cross-scale information and preservation of critical semantic features. This structure facilitates the construction of compact yet rich feature representations, significantly reduces computational overhead, and improves the model’s processing efficiency and representational capacity in complex scenarios. The mathematical formulation of the RMP module is presented in Equation (12):

(12) $$\begin{array}{l} input_{RMP}=output_{DAC}=Y\in R^{C\times H\times W}\\ P_{1}(Y)=Maxpool_{2\times 2}(Y)\\ P_{2}(Y)=Maxpool_{3\times 3}(Y)\\ P_{3}(Y)=Maxpool_{5\times 5}(Y)\\ P_{4}(Y)=Maxpool_{6\times 6}(Y)\\ U_{i}{\left(Y\right)}_{i=1,2,3,4}=Upsample(Conv_{1\times 1}(P_{i}(Y)))\\ output_{RMP}=Concat(U_{1}(Y),U_{2}(Y),U_{3}(Y),U_{4}(Y),Y) \end{array}$$

where $Maxpool_{n\times n}$ denotes max pooling operations with different kernel sizes, Upsample represents the upsampling operation, and Concat indicates the feature concatenation operation.

### 2.6. Rationale for Model Design

The D-LKA module combines deformable convolution with large kernel attention to enhance the modeling of long-range dependencies and spatial context. In liver tumor segmentation, where tumors often exhibit irregular shapes and low contrast with the liver parenchyma, the choice of kernel size largely determines the scope and granularity of feature integration. Small kernels are more effective in capturing local details and boundary information, but they lack sufficient receptive fields to establish global connections for large or diffuse tumors. Conversely, large kernels expand the receptive field and enrich contextual information, but they may also introduce irrelevant background noise that weakens discriminative capability.

The CE module typically employs convolution kernels of varying sizes to construct multi-scale feature extraction pathways. An appropriate combination of kernel sizes is crucial for accurately segmenting tumors of different scales while delineating their boundaries. Smaller kernels are better suited for capturing fine structural details at tumor edges, whereas larger kernels facilitate the modeling of the overall spatial distribution of tumors and their relationship with the surrounding liver parenchyma, thereby balancing local and global information.

The DCA mechanism establishes dynamic non-local connections across feature map positions via attention, while its deformable property enables the model to adaptively focus on context regions most relevant to tumor pixels, overcoming the limitations of fixed geometric structures. In liver tumor segmentation, the number of attention heads determines the model’s ability to capture diverse feature relationships in parallel. Increasing the number of heads allows the model to learn multiple representation patterns, such as intra-tumor texture heterogeneity, proximity to vessels, and overall boundary constraints, thereby improving segmentation accuracy for complex tumors. However, more heads do not necessarily yield better performance: too few may lead to insufficient context modeling, whereas too many increase the risk of overfitting and may interfere with deformable offset prediction. Thus, the number of heads must be carefully balanced between feature diversity and model generalization.

The proposed model integrates three key modules for attention and feature enhancement. The D-LKA (Deformable Large Kernel Attention) employs two deformable convolution layers with kernel sizes of 5 × 5 and 7 × 7, where the 7 × 7 kernel is combined with dilation (dilation = 3) and corresponding padding (padding = 9) to enlarge the receptive field without compromising resolution, ensuring that the output feature map size matches the input. The DAC (Dense Atrous Convolution) module utilizes parallel 3 × 3 convolutions with dilation rates of 1, 3, and 5 to capture multi-scale contextual information, further combined with the RMP (Residual Multi-kernel Pooling) module, which extracts global features through pooling at four scales (2 × 2, 3 × 3, 5 × 5, and 6 × 6). After upsampling and concatenation, the bottleneck channel dimension expands from 512 to 516 (512 + 4), yielding an output of 516 × 32 × 32 while only slightly increasing the parameter count. The DCA (Dual Cross Attention) mechanism is configured with one channel attention head (channel_head = 1) and four spatial attention heads (spatial_head = 4) to facilitate interaction and enhancement of encoder features. The spatial resolution remains unchanged before and after processing, ensuring effective fusion with decoder skip connections.

The input image (3 × 512 × 512) is progressively processed by the encoder. Initially, convolution and D-LKA are applied to extract 64 × 512 × 512 feature maps, followed by max-pooling that downsamples them to 64 × 256 × 256. This process is repeated across successive layers, with the number of channels doubling and the spatial resolution halving at each stage, generating feature maps of 128 × 256 × 256, 256 × 128 × 128, and 512 × 64 × 64, and finally downsampling to 512 × 32 × 32. At the bottleneck, DAC combined with RMP expand the channel dimension to 516 × 32 × 32, thereby enriching contextual information.

The DCA module enhances features across encoder layers while preserving their original dimensions (ranging from 64 × 512 × 512 to 512 × 64 × 64). The decoder then progressively restores spatial resolution through upsampling. Starting from 516 × 32 × 32, the feature maps are upsampled to 512 × 64 × 64 and fused with corresponding encoder features via concatenation. This process reconstructs feature maps of 256 × 128 × 128, 128 × 256 × 256, and 64 × 512 × 512, before the final convolution produces a 2 × 512 × 512 segmentation output, fully recovering the input resolution.

## 3. Evaluation Metrics for Segmentation Performance

To comprehensively evaluate the model’s performance in liver tumor semantic segmentation, seven quantitative metrics were employed: Precision, Recall, Dice Similarity Coefficient (Dice), Intersection over Union (IoU), mean Intersection over Union (MIoU), Accuracy, and Mean Pixel Accuracy (MPA). True Positive (TP) refers to the number of pixels correctly predicted as a liver tumor by the model. This indicates the model’s ability to accurately identify tumor regions that are truly present. False Positive (FP) denotes the number of non-tumor pixels incorrectly classified as tumor by the model. This reflects false alarms where the model erroneously labels healthy tissue as tumor. False Negative (FN) indicates the number of tumor pixels mistakenly predicted as non-tumor. This reveals instances where the model fails to detect actual tumor regions. True Negative (TN) represents the number of non-tumor pixels correctly identified as such by the model. This demonstrates the model’s capacity to correctly exclude regions that do not contain tumors.

Precision refers to the proportion of correctly identified liver tumor samples among all samples predicted by the model as liver tumors. It reflects the reliability of the model’s positive predictions. The formulation is presented in Equation (13):

(13) $$P=\frac{TP}{TP+FP}$$

Recall refers to the proportion of correctly detected liver tumor samples among all actual tumor samples. It measures the model’s ability to identify positive instances. The formulation is provided in Equation (14):

(14) $$R=\frac{TP}{TP+FN}$$

The Dice Similarity Coefficient (Dice) is used to evaluate the overlap between the predicted liver tumor region and the ground truth annotation. It is defined as twice the area of the intersection divided by the sum of the areas of the predicted and ground truth regions. The formulation is presented in Equation (15):

(15) $$Dice=\frac{2\times TP}{2\times TP+FP+FN}$$

The Intersection over Union (IoU) is another commonly used overlap metric, defined as the ratio of the intersection area to the union area between the predicted liver tumor region and the ground truth. Compared with the Dice coefficient, IoU adopts a more stringent formulation, and thus typically yields lower values under the same conditions. The formulation is presented in Equation (16):

(16) $$IoU=\frac{TP}{FN+FP+TP}$$

The mean Intersection over Union (*MIoU*) is defined as the average IoU across all classes and is used to comprehensively evaluate the model’s segmentation performance for each class. For liver tumor semantic segmentation, which is a binary classification task, the formulation is given in Equation (17):

(17) $$MIoU=\frac{\frac{TP}{FN+FP+TP}+\frac{TN}{FN+FP+TN}}{2}$$

Accuracy represents the proportion of correctly predicted pixels to the total number of pixels. The formulation is provided in Equation (18):

(18) $$Accuary=\frac{TP+TN}{TP+TN+FP+FN}$$

Mean Pixel *Accuracy* (MPA) refers to the average accuracy with which the model correctly classifies pixels across all classes. The formulation is given in Equation (19):

(19) $$MPA=\frac{1}{k+1}\frac{\sum_{i=0}^{k}p_{ii}}{\sum_{i=0}^{k}\sum_{j=0}^{k}P_{ij}}$$

where *k* denotes the number of classes; *p_ii_* represents the number of pixels that actually belong to class *i* and are correctly predicted as class *i*, while *P_ij_* denotes the number of pixels that belong to class *i* but are incorrectly predicted as class *j*.

## 4. Datasets and Experimental Design

Currently, three publicly available liver tumor imaging datasets are widely used: the LiTS dataset from the Liver Tumor Segmentation Challenge 2017 Database, the 3D-IRCADb01 dataset from the 3D Image Reconstruction for Comparison of Algorithm Database, and the MSD Task08 dataset from the Medical Segmentation Decathlon Task08 Database.

The LiTS dataset is a CT imaging dataset specifically designed for liver tumor segmentation. It comprises 3D CT scans collected from multiple medical centers, including 131 cases for training and 70 for testing. The 3D-IRCADb01 dataset consists of 3D CT scans from 20 patients (10 female and 10 male), the majority of whom have liver tumors. It contains 20 folders, each corresponding to an individual patient. Among the various annotated structures, the ground truth for liver tumors is selected for analysis. The MSD Task08 dataset includes 443 3D CT volumes, divided into 303 training cases and 140 testing cases.

To ensure annotation accuracy, all datasets were reverified and corrected by experienced radiologists.

**Figure 7:**
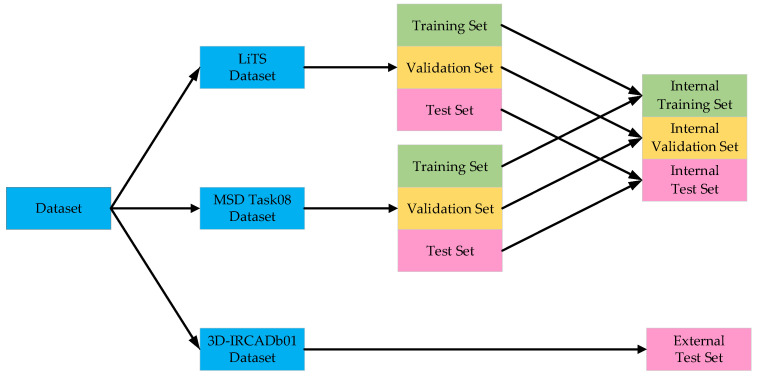
Dataset partitioning results.

In the aforementioned liver tumor datasets, the test sets do not provide ground truth annotations for liver tumors; therefore, they were excluded from the experimental samples. Specifically, the LiTS dataset contains 118 annotated tumor cases, the 3D-IRCADb01 dataset includes 15 annotated cases, and the MSD Task08 dataset provides 303 annotated cases. To avoid evaluation bias caused by data leakage, the training, validation, and test sets were constructed based on patient-level partitioning. The LiTS and MSD Task08 datasets were each divided into training, validation, and test sets using an 8:1:1 ratio. The training sets from both datasets were merged, and the same strategy was applied to the validation and test sets. Furthermore, given the significantly lower number of tumor cases compared to normal cases in the datasets, non-tumor cases were excluded from the training, validation, and test sets to mitigate the impact of class imbalance and enhance the model’s ability to learn liver tumor features. To evaluate the generalization capability of the proposed model, the 3D-IRCADb01 dataset was used as an independent external test set. The dataset partitioning scheme and detailed results are illustrated in Figure 7 and Table 1.

To improve the model’s generalizability across multi-source datasets and harmonize spatial scale differences among LiTS, MSD Task08, and 3D-IRCADb01—which exhibit substantial variability in scanning parameters such as slice thickness and voxel spacing—all images were resampled to a uniform voxel resolution. This standardization ensures consistent spatial resolution of the liver region across all images, thereby minimizing distribution discrepancies caused by differences in scanning equipment and centers.

To enhance the effectiveness and compatibility of image representation, and considering that the typical Hounsfield Unit (HU) range for liver CT images lies between [40, 70], this study truncates the CT image HU values to the interval of [−400, 400]. The images are first clipped to this range, followed by linear normalization to map the pixel values to the [0, 255] scale. Subsequently, the data is converted to uint8 format to meet the input data type and value range requirements of the neural network. Finally, the processed images are saved in JPG format, with corresponding annotations stored as PNG files.

To improve model robustness and mitigate potential overfitting caused by the limited number of training samples, various data augmentation strategies were employed during training. These included random rotations (±15°) and random scaling (with a scale factor ranging from 0.9 to 1.1), which effectively increased sample diversity without significantly compromising the structural integrity of the original images. It is important to note that data augmentation was applied only to the training set, while the validation and test sets were kept unchanged to ensure the objectivity and comparability of model evaluation results.

To ensure the reproducibility and reliability of the liver tumor semantic segmentation experiments, all experiments were conducted on a carefully configured hardware and software platform. The hardware environment consisted of a workstation running Ubuntu 22.04, equipped with an Intel Core i7-9700K processor (3.80 GHz), an NVIDIA GeForce GTX 2080 Ti GPU, and 32 GB of RAM, which collectively met the computational and memory demands of deep learning model training and inference. The software environment was based on the PyTorch 1.12.1 deep learning framework and Python 3.7.13 for model development and training. CUDA 11.6 and cuDNN 8.5.x libraries were integrated to fully leverage GPU parallelism and significantly accelerate the training process.

Regarding hyperparameter settings, the model was trained for 50 epochs with a batch size of 4. A cosine annealing learning rate schedule was employed, starting from 1 × 10^−4^ and gradually decreasing to 1 × 10^−7^. This strategy enables rapid convergence in the early training stages while helping to prevent overfitting in later epochs. The Adam optimizer, known for its efficient adaptive learning rate mechanism, was adopted to facilitate fast and stable model convergence. Details of the experimental environment and hyperparameter configurations are summarized in Table 2.

## 5. Loss Function Design

In the semantic segmentation task of liver tumors, Focal Loss and Dice Loss were adopted as the primary loss functions to guide network optimization during training.

Focal Loss is a loss function specifically designed to address the issue of class imbalance. In liver tumor segmentation tasks, the tumor regions typically occupy only a small portion of the entire image, resulting in a severely imbalanced class distribution that negatively impacts model training. To mitigate this issue, Focal Loss was introduced as the primary supervised loss function. By introducing a modulating factor, Focal Loss effectively down-weights easy-to-classify examples, encouraging the model to focus more on hard-to-classify samples. This mechanism alleviates class imbalance and enhances the model’s attention to informative examples. Moreover, Focal Loss emphasizes low-confidence and difficult samples, which helps the model better attend to boundary regions and hard-to-segment areas, thereby improving its ability to learn and represent task-critical regions. The formulation of Focal Loss is given in Equation (20):

(20) $$FL(p_{t})=-{\left(1-p_{t}\right)}^{\gamma }\log(p_{t})$$

where $p_{t}$ denotes the predicted probability for the true class. In the context of liver tumor segmentation, if the current pixel belongs to the tumor class, then $p_{t}$ represents the confidence score for the tumor; otherwise, $p_{t}$ indicates the confidence for the background. γ is the modulating factor. Setting γ to 1 effectively enhances the model’s sensitivity in identifying tumor regions, particularly for lesions with indistinct boundaries or small volumes.

Dice Loss is a widely used loss function in image segmentation tasks that evaluates model performance by measuring the similarity between the predicted and ground truth regions. In liver tumor segmentation, Dice Loss quantifies the spatial overlap between the model’s segmentation output and the true tumor region. Compared to loss functions such as cross-entropy, Dice Loss is more robust to class imbalance, as it focuses on the overlap of regions rather than individual sample classes. The formulation of Dice Loss is presented in Equation (21):

(21) $$DL(p,g)=1-Dice=1-\frac{2\times \sum_{i}(p_{i}\times g_{i})+\varepsilon }{\sum_{i}p_{i}^{2}+\sum_{i}g_{i}^{2}+\varepsilon }$$

where p represents the model’s prediction for liver tumor pixels, g denotes the ground truth labels of the tumor pixels, and ε is a smoothing term, typically set to a small positive constant to prevent numerical instability caused by division by zero.

In the semantic segmentation task of liver tumors, a combined use of Focal Loss and Dice Loss is employed. This approach addresses class imbalance while emphasizing accurate segmentation boundary alignment. Typically, the two losses are summed to form a composite objective function, as expressed in Equation (22):

(22) $$L_{T}=L_{F}+\lambda L_{D}=-{\left(1-p_{t}\right)}^{\gamma }\log(p_{t})+\lambda (1-\frac{2\times \sum_{i}(p_{i}\times g_{i})+\varepsilon }{\sum_{i}p_{i}^{2}+\sum_{i}g_{i}^{2}+\varepsilon })$$

where λ is set to 1, which balances class imbalance handling and region matching accuracy without introducing additional tuning complexity.

## 6. Experimental Results and Performance Analysis

### 6.1. Ablation Experiments Analysis

To validate the effectiveness of the proposed liver tumor semantic segmentation method, an ablation study was conducted in this section. Specifically, D1 denotes the Deformable Large Kernel Attention module, C represents the Context Extraction mechanism, D2 stands for the Dual Cross Attention mechanism, and D1CD2U-Net refers to the proposed segmentation network. Modules were progressively ablated to analyze their individual contributions to liver tumor segmentation performance. Furthermore, seven segmentation metrics—Precision, Recall, Dice, IoU, MIoU, Accuracy, and MPA—were employed to evaluate the algorithm’s performance. The detailed ablation results are presented in Table 3.

To evaluate the impact of the Deformable Large Kernel Attention module on liver tumor segmentation performance, an ablation study was conducted by removing this module and observing changes in segmentation metrics. The results indicate that, compared to the full D1CD2U-Net, the CD2U-Net without the Deformable Large Kernel Attention module exhibits decreases in Precision, Recall, and IoU, reflecting a notable decline in the model’s ability to extract liver tumor features. This demonstrates that the Deformable Large Kernel Attention mechanism plays a critical role in the model and significantly contributes to liver tumor segmentation performance.

To assess the effect of the Dual Cross Attention mechanism on liver tumor segmentation, an ablation study was performed by progressively removing this module and monitoring changes in performance metrics. The ablation results reveal that, relative to D1CD2U-Net, the D1CU-Net—lacking the Dual Cross Attention module—shows reductions in Precision, Recall, and IoU, indicating certain limitations in feature fusion. This suggests that incorporating the Dual Cross Attention mechanism effectively enhances the integration of deep and shallow features within the model. To evaluate the impact of the Context Extraction mechanism on liver tumor segmentation, an ablation study was conducted by progressively removing this module and observing the corresponding changes in performance metrics. The results show that, compared with D1CD2U-Net, the D1D2U-Net variant exhibits decreased Precision, Recall, and IoU, reflecting a reduced capability to capture global information. These findings indicate that the Context Extraction mechanism is beneficial for this task and contributes to improved model performance in liver tumor segmentation.

Overall, the ablation study thoroughly validates the significant contribution of the Deformable Large Kernel Attention module, Context Extraction mechanism, and Dual Cross Attention mechanism in enhancing liver tumor segmentation performance. The experimental results demonstrate that D1CD2U-Net achieves superior performance across all evaluation metrics, further confirming the critical roles of these modules in feature representation and information integration, as well as their synergistic effects. Specifically, Deformable Large Kernel Attention enhances feature extraction, effectively capturing fine-grained structural details of the liver and tumor regions; the Context Extraction module focuses on global semantic modeling, providing comprehensive contextual information that allows the model to accurately identify tumor regions even in complex backgrounds; and the Dual Cross Attention module strengthens local feature representation through cross-channel feature fusion, improving sensitivity to edge details and small lesions. Although Context Extraction and Dual Cross Attention exhibit functional complementarity, their overlap is limited—for instance, Context Extraction can also provide additional information enhancement in multi-scale feature interactions. Therefore, Context Extraction and Dual Cross Attention are not entirely independent but display significant synergy: global semantic guidance from Context Extraction enables Dual Cross Attention to perform more precise local feature fusion, collectively enhancing the model’s segmentation accuracy, stability, and adaptability to complex tumor morphologies.

### 6.2. Performance Comparison with State-of-the-Art Methods

#### 6.2.1. Performance Comparison on the Internal Test Set

To demonstrate the performance advantages of the proposed liver tumor semantic segmentation method, this study conducted a comprehensive comparison between D1CD2U-Net and nine state-of-the-art network architectures, including U-Net, FCN, LinkNet, PSPNet, DeeplabV3+, Unext, AG-Net, MISSFormer, and TransCeption. Consistent with the ablation study, seven segmentation metrics—Precision, Recall, Dice, IoU, MIoU, Accuracy, and MPA—were utilized to evaluate the algorithm’s performance. The detailed comparison results are presented in Table 4.

In the field of liver tumor segmentation, numerous classical network architectures—such as U-Net, FCN, LinkNet, PSPNet, DeeplabV3+, Unext, AG-Net, MISSFormer, and TransCeption—have been proposed, each incorporating distinctive feature extraction and fusion strategies aimed at improving segmentation accuracy. U-Net achieves deep multi-scale feature fusion through skip connections and feature concatenation. FCN and LinkNet employ feature weighting strategies to optimize feature integration. PSPNet introduces a pyramid pooling module to enhance the integration of global contextual information. DeeplabV3+ leverages atrous spatial pyramid pooling to expand the receptive field for contextual awareness. Unext improves sensitivity to local features through multilayer perception-based designs. AG-Net enhances the response to critical features by combining attention mechanisms with structure-preserving techniques. MISSFormer and TransCeption adopt Transformer-based architectures, which significantly enhance the model’s global modeling capability.

Although current mainstream models have achieved notable progress in liver tumor segmentation, the evaluation metrics presented in Table 4 reveal limitations in both feature fusion and feature extraction across these architectures. The feature fusion mechanisms in U-Net, FCN, LinkNet, and AG-Net struggle to effectively bridge the semantic gap between shallow and deep features. The feature extraction strategies employed by all the comparative models remain insufficient for capturing the complex and fine-grained characteristics of tumor regions, thereby limiting their ability to achieve highly precise segmentation. While PSPNet, DeeplabV3+, and Unext demonstrate effective deep feature extraction, they are limited in their ability to capture broader global contextual information. MISSFormer and TransCeption focus primarily on global context modeling but tend to neglect the extraction of local details. In response to these challenges, this study proposes the D1CD2U-Net model, which incorporates a series of carefully designed enhancements to address the aforementioned limitations.

In the encoder stage of the model, a Deformable Large Kernel Attention mechanism is effectively integrated. This module is designed to enhance the network’s sensitivity to tumor regions while mitigating the loss of critical features during the downsampling process, thereby enabling more accurate and efficient capture of tumor-related features. Furthermore, a Context Extraction mechanism is introduced at the bottleneck layer. By expanding the receptive field and integrating multi-scale information, this module significantly improves the network’s ability to capture global contextual features, providing richer and more comprehensive support for subsequent segmentation tasks. In addition, the conventional skip connection strategy is replaced with a Dual Cross Attention mechanism. This mechanism not only facilitates effective communication and fusion between features at different scales—thereby alleviating the semantic gap—but also greatly enhances the network’s global modeling capability. As a result, the model is able to make more accurate and robust segmentation decisions when dealing with complex and heterogeneous liver tumor images.

According to the evaluation metrics, the proposed D1CD2U-Net consistently outperforms competing models in terms of Precision, Recall, Dice, IoU, MIoU, Accuracy, and MPA. A high Precision score indicates the model’s strong accuracy in identifying tumor regions, while a high Recall reflects its high sensitivity in detecting tumor instances. The high Dice coefficient further validates the model’s superior performance in segmenting tumor regions. As the harmonic mean of Precision and Recall, Dice reflects both the sensitivity and consistency of the model in identifying overlapping tumor regions, highlighting its effectiveness in capturing local structural accuracy. The elevated values of IoU and MIoU indicate the model’s spatial coverage accuracy in both binary and multi-class segmentation tasks. Specifically, IoU quantifies the ratio between the intersection and union of predicted and ground truth regions, emphasizing the model’s exactness in covering target areas. As the average IoU across all classes, MIoU reflects the model’s consistency and generalization capability across various categories. The high values of Accuracy and MPA further demonstrate the model’s classification performance at both global and pixel levels. The combined performance across all metrics confirms the efficiency and accuracy of the D1CD2U-Net in liver tumor segmentation tasks. In particular, the jointly high values of Dice and IoU demonstrate that the model can not only accurately localize tumor regions but also maintain strong agreement with manual annotations—an essential attribute for supporting precision diagnosis and clinical decision-making.

#### 6.2.2. Performance Comparison on the External Test Set

To comprehensively and rigorously evaluate the generalization capability of the D1CD2U-Net model, the 3D-IRCADb01 dataset was selected as an independent test benchmark. This choice aims to provide an accurate assessment of the model’s real-world effectiveness when applied to unseen and previously unencountered data, thereby avoiding potential biases associated with the original test set. The detailed comparison results are presented in Table 5.

D1CD2U-Net exhibits competitive performance across multiple key performance metrics. Specifically, it achieved a Precision of 0.9607, significantly outperforming all comparative models, indicating high consistency between predicted tumor regions and ground truth. The model also attained a Recall of 0.7421, reflecting its ability to identify most actual liver tumor regions. Although this score suggests further improvement is possible, D1CD2U-Net surpasses other models in reducing false negatives.

Notably, its Dice score reached 0.8376. As a metric evaluating overlap between predicted and annotated regions, this highlights the model’s sensitivity to tumor boundaries and morphological details. Additionally, the model achieved an IoU of 0.7203, directly quantifying segmentation accuracy. The MIoU of 0.8587 further confirms its high accuracy and consistency for all classes.

D1CD2U-Net also attained the Accuracy of 0.9972, demonstrating exceptional classification performance. These results reflect the model’s robustness in liver tumor segmentation and its capability to handle complex medical imaging data. With an MPA of 0.8709, it also achieves high pixel-level classification precision. In summary, D1CD2U-Net exhibits superior segmentation performance and generalization ability for liver tumor segmentation tasks.

### 6.3. Saliency Analysis of the Model

**Figure 8:**
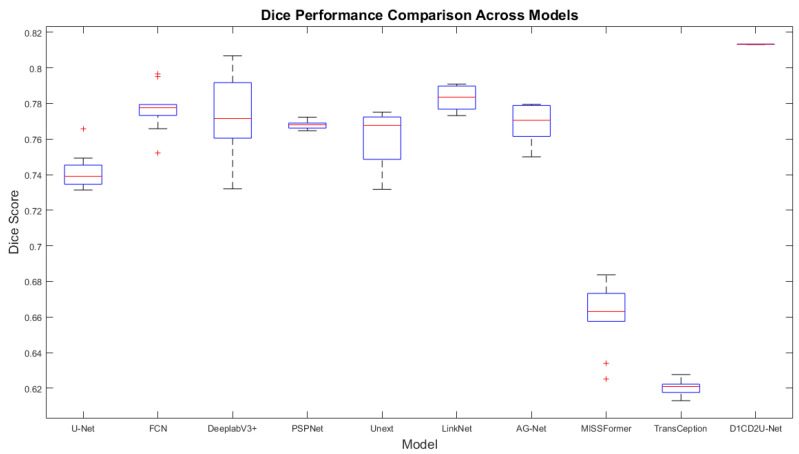
Boxplot of Dice scores on the internal test set.

**Figure 9:**
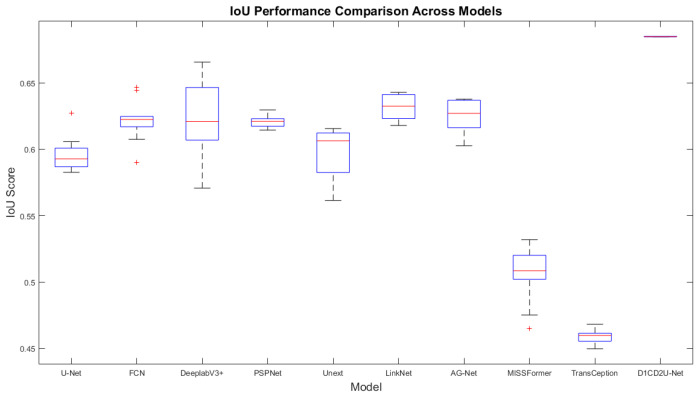
Boxplot of IoU scores on the internal test set.

**Figure 10:**
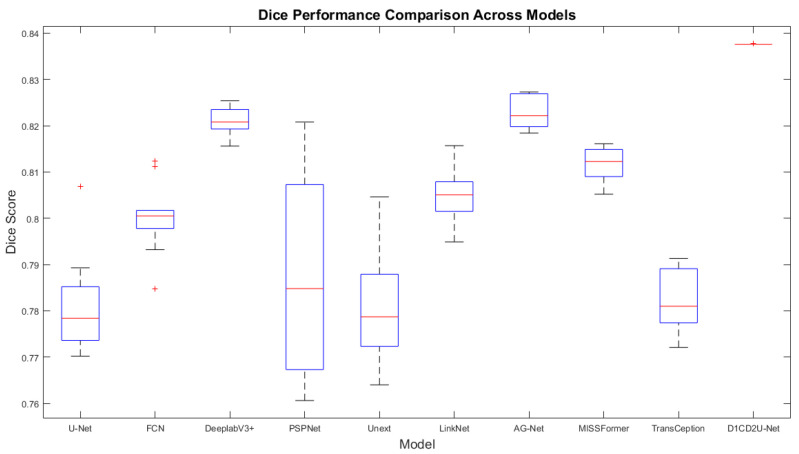
Boxplot of Dice scores on the external test set.

**Figure 11:**
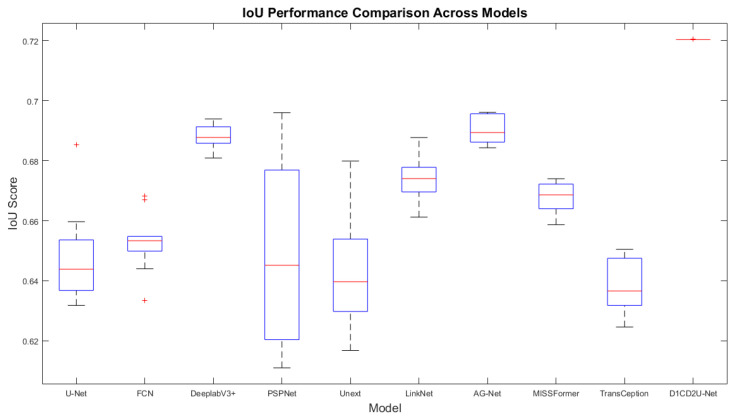
Boxplot of IoU scores on the external test set.

Most evaluation metrics currently report only average performance, lacking statistical verification of method improvements. To quantify the performance gains of the proposed approach relative to other models, this section employs the paired *t*-test to assess the significance of differences using *p*-values, accompanied by boxplots for visual illustration, thereby enhancing the reliability and interpretability of the results. The red markers in the box plot represent outliers. The specific experimental results are presented in Table 6 and Table 7, as well as Figure 8, Figure 9, Figure 10 and Figure 11. All model performance metrics are reported as the mean ± standard deviation across 10 independent experiments. A two-tailed paired *t*-test was used to compare each model against the improved model (D1CD2U-Net). An asterisk (*) indicates that the performance difference relative to the improved model is statistically significant (*p* < 0.05), and this explanation is applicable to both Table 6 and Table 7. 

In the task of liver tumor semantic segmentation, the performance of different models on the internal test set showed significant variation. The proposed D1CD2U-Net achieved the highest performance in both Dice coefficient and IoU, significantly outperforming all comparison models (*p* < 0.05). Traditional CNN models demonstrated stable performance, with Dice scores ranging from 0.75 to 0.77 and IoU around 0.61–0.62, indicating their ability to capture tumor boundaries and regional features. However, their overall performance shows clear limitations. Among them, AG-Net, which incorporates attention mechanisms, performed best among traditional architectures, suggesting that attention modules can enhance segmentation accuracy. Some Transformer-based models performed poorly, with Dice scores below 0.70, likely due to their high data requirements and tendency to underfit with limited datasets. The lightweight model Unext also failed to achieve competitive performance, highlighting the trade-off between model complexity and representational capacity. D1CD2U-Net, through the integration of DCA, D-LKA, and CE modules, enhanced sensitivity to fine structures and edges, achieving higher segmentation accuracy while demonstrating superior stability. Boxplot analysis further confirmed that D1CD2U-Net not only achieved the best performance but also exhibited the smallest variability, reflecting its outstanding stability.

On the external test set, model performance in liver tumor semantic segmentation showed trends similar to those observed on the internal test set. The proposed D1CD2U-Net continued to significantly outperform all comparison models in both Dice coefficient and IoU (*p* < 0.05), demonstrating excellent generalization ability. Traditional CNN models such as DeeplabV3+, LinkNet, and AG-Net exhibited relatively stable performance on external data, with Dice scores ranging from 0.80 to 0.81 and IoU around 0.67–0.68. Among these, AG-Net remained the best-performing conventional architecture, further confirming the benefits of attention mechanisms for handling complex scenarios. Some models, including U-Net, FCN, and Unext, showed slight improvements on the external test set but still performed significantly worse than the improved model. Transformer-based models such as MISSFormer and TransCeption continued to underperform, with Dice scores below 0.79, indicating limited generalization across differing data distributions. Boxplot analysis further revealed that D1CD2U-Net not only achieved the highest median performance but also exhibited minimal interquartile range, reflecting outstanding stability and generalization capability. Thanks to its specialized module design, D1CD2U-Net maintained optimal performance with minimal variability on external datasets, further highlighting its robustness and practical value in complex clinical scenarios.

### 6.4. Analysis of Model Complexity and Computational Cost

In liver tumor semantic segmentation, model computational complexity and time cost are critical factors in addition to segmentation accuracy. High-performance models often involve larger parameter counts and increased computations, which can lead to inference delays or higher resource consumption, affecting deployability and real-time applicability. Therefore, analyzing the number of parameters, FLOPs, training time, and inference time for the proposed and comparison models helps to comprehensively assess the balance between performance and efficiency, providing guidance for clinical deployment. The specific details are shown in Table 8. It is important to note that Inference time 1 refers to the internal test set, whereas Inference time 2 refers to the external test set.

In liver tumor semantic segmentation, although D1CD2U-Net has significantly higher parameter counts and computational complexity than lightweight networks, this complexity represents a key advantage. The larger model capacity enables the network to learn richer feature representations and more complex spatial semantic information, effectively capturing fine structural details of the liver and tumor regions, thereby enhancing segmentation accuracy and robustness. This is particularly critical for clinical images with highly variable tumor morphology and irregular boundaries. Compared to conventional models, D1CD2U-Net demonstrates superior capabilities in global context modeling and multi-scale feature fusion, providing more stable segmentation results under complex lesion conditions.

In terms of time efficiency, although training D1CD2U-Net requires longer durations, the inference time per CT slice is approximately 0.1032 s. While slightly higher than other large networks, this remains within a clinically acceptable range. Considering that liver tumor segmentation is typically performed as an offline auxiliary diagnostic task, training time can be optimized through high-performance computing or distributed training, while inference time fully meets clinical application requirements. Therefore, D1CD2U-Net achieves a favorable balance between high-capacity feature learning and computational efficiency, providing a solid foundation for precise liver tumor segmentation.

### 6.5. Feature Activation Map Comparison Between the Baseline Network and the Proposed Model

**Figure 12:**
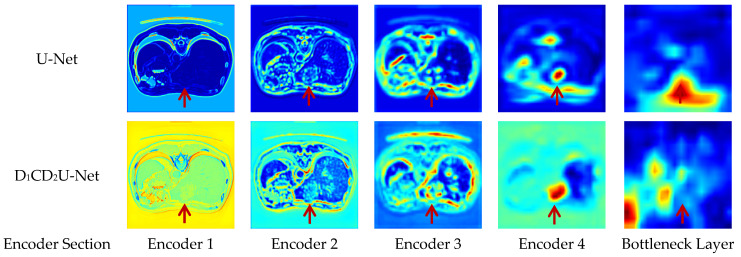
Comparison of Feature Activation Maps Between U-Net and the proposed model in encoder and bottleneck layers.

**Figure 13:**
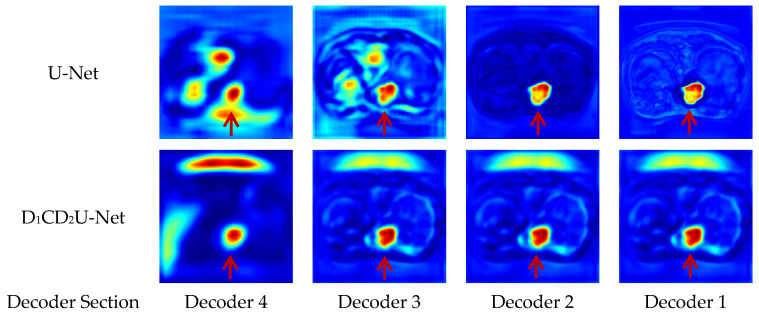
Comparison of Feature Activation Maps between U-Net and the proposed model in decoder layers.

To gain deeper insight into the internal mechanisms of the model architectures and enhance their interpretability, this section presents activation heatmaps for U-Net and D1CD2U-Net applied to the image segmentation task. Specifically, the comparison focuses on changes in activated regions during feature extraction and reconstruction, aiming to reveal performance differences through intuitive visualization. The first row displays segmentation heatmaps of the U-Net architecture, while the second row shows those of D1CD2U-Net. Columns one through four correspond to the encoder heatmaps, column five represents the bottleneck layer, and columns six through ten display decoder heatmaps. The detailed heatmap visualizations are presented in Figure 12 and Figure 13.

From Encoder Layer 1 to Layer 4, as downsampling operations proceed, U-Net progressively extracts higher-level features from the images; however, this process inevitably results in the loss of some spatial information. This issue is particularly pronounced when segmenting liver tumors, which occupy a relatively small portion of the image; their distinctive texture and shape features tend to become increasingly blurred during encoding. At the bottleneck layer, U-Net further compresses the features extracted by the encoder to prepare for the decoding phase. However, due to the earlier loss of features in preceding layers, the representations at the bottleneck may no longer sufficiently capture all the fine-grained details of the liver tumors. In the decoder layers, U-Net attempts to restore spatial information through upsampling and skip connections. Nevertheless, owing to information loss during encoding, the decoder faces significant challenges in recovering tumor details, which can lead to incomplete or blurred liver tumor regions in the final segmentation results.

Compared to U-Net, D1CD2U-Net demonstrates enhanced feature extraction capabilities at the encoder stage. This improvement primarily results from the integration of the Deformable Large Kernel Attention mechanism, which more effectively extracts and strengthens critical features within the image, especially for liver tumors that are relatively scarce and embedded in complex backgrounds. Consequently, by Encoder Layer 4, D1CD2U-Net is able to accurately identify and reinforce tumor-specific features. At the bottleneck layer, D1CD2U-Net performs crucial feature compression and integration, effectively consolidating the rich and precise features extracted by the encoder, thereby laying a solid foundation for the subsequent decoding process. Given the high quality of encoder features, the integrated features at the bottleneck comprehensively and accurately represent the complete information of liver tumors. To further enhance global information comprehension, a Context Extraction mechanism is incorporated at the bottleneck layer, significantly improving the model’s ability to capture and interpret global context.

Meanwhile, to promote effective fusion between features at different levels and prevent information loss, D1CD2U-Net replaces conventional skip connections with a Dual Cross Attention mechanism. This design not only ensures smooth transmission of features across levels but also facilitates deep fusion and complementation between features, achieving more efficient and precise integration while further enhancing the model’s global modeling capability. During decoding, the synergistic effect of the Context Extraction and Dual Cross Attention mechanism greatly improves spatial detail restoration. The Context Extraction mechanism precisely guides spatial position reconstruction during decoding, ensuring accurate localization of liver tumors. The Dual Cross Attention mechanism meticulously restores tumor details, enabling the network to increasingly focus on tumor regions from Decoder Layer 4 to Layer 1. The deeper coloration of tumor regions in output images directly reflects the model’s high confidence in tumor identification.

### 6.6. Segmentation Results Visualization and Analysis

#### 6.6.1. Segmentation Visualization on the Internal Test Set

**Figure 14:**
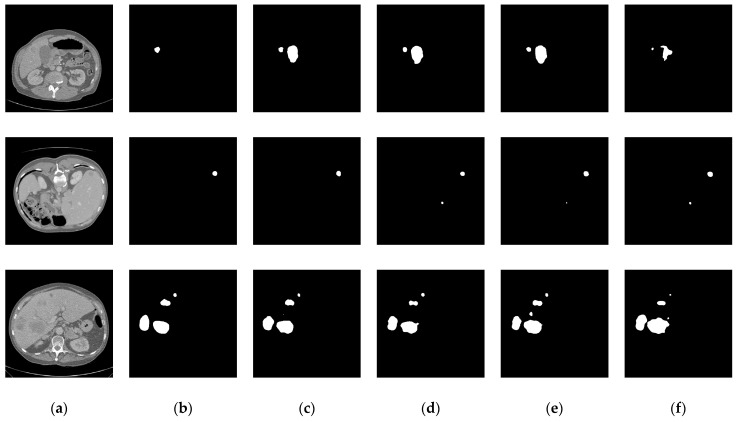
Comparative segmentation results on the internal test set; (**a**) original image; (**b**) ground truth; (**c**) U-Net; (**d**) FCN; (**e**) LinkNet; (**f**) Unext.

**Figure 15:**
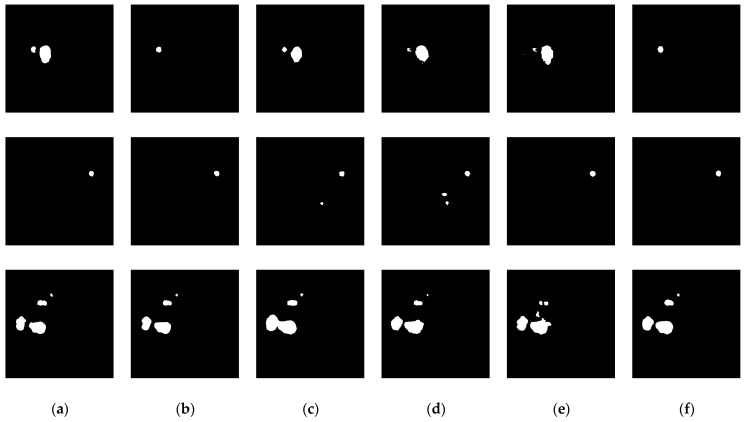
Comparative segmentation results on the internal test set; (**a**) AG-Net; (**b**); DeeplabV3+; (**c**) PSPNet; (**d**) MISSFormer; (**e**) TransCeption; (**f**) D1CD2U-Net.

To more intuitively demonstrate the superiority of the proposed model in liver tumor segmentation, this section presents a comparative analysis of segmentation results from various models. In Figure 14, panel (a) shows the original liver tumor images, (b) represents the ground truth annotations, while panels (c) through (f) display segmentation results generated by U-Net, FCN, LinkNet, and Unext, respectively. Figure 15 illustrates the segmentation outcomes of AG-Net, DeeplabV3+, PSPNet, MISSFormer, and TransCeption in panels (**a**–**e**), with panel (f) presenting the results of the proposed D1CD2U-Net model. The detailed segmentation results are presented in Figure 14 and Figure 15. The original images in Figure 14, from top to bottom, correspond to Example 1, Example 2, and Example 3, respectively.

In the first and second rows, the liver tumors are located in the upper left and upper right corners of the images, respectively. These cases involve single and small-sized tumor regions. Except for DeeplabV3+, all other models exhibit clear over-segmentation, which may lead to a higher false positive rate. In contrast, the proposed model demonstrates superior accuracy and robustness when segmenting these small and spatially diverse tumors, effectively avoiding over-segmentation and thereby confirming its advantage in small-object segmentation tasks.

Furthermore, when dealing with multiple, spatially separated liver tumor regions—as illustrated in the third row—the complexity of the segmentation task increases significantly. In this scenario, AG-Net and DeeplabV3+ tend to under-segment the tumors, potentially missing some tumor areas and resulting in false negatives. Other models generally suffer from over-segmentation, introducing false positives. In contrast, the proposed model accurately identifies and segments each distinct tumor region, effectively reducing both false positives and false negatives. This highlights its strong performance in handling complex tumor distributions.

In summary, the comparative analysis clearly demonstrates that the proposed model outperforms other approaches in terms of segmentation accuracy, robustness, and adaptability to complex scenarios. Notably, the model exhibits significant advantages in segmenting small tumors and multiple non-contiguous tumor regions.

#### 6.6.2. Segmentation Visualization on the External Test Set

**Figure 16:**
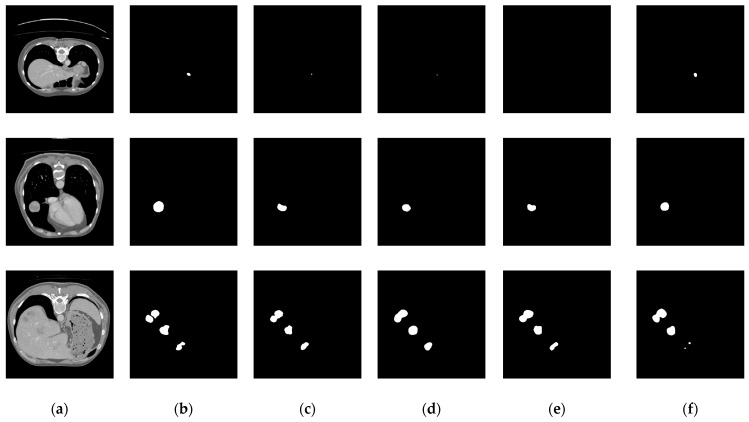
Comparative segmentation results on the external test set; (**a**) original image; (**b**) ground truth; (**c**) U-Net; (d) FCN; (**e**) LinkNet; (**f**) Unext.

**Figure 17:**
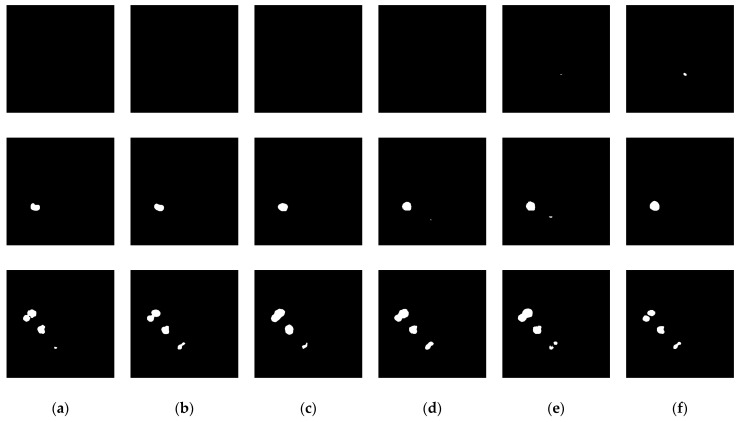
Comparative segmentation results on the external test set; (**a**) AG-Net; (**b**) DeeplabV3+; (**c**) PSPNet; (**d**) MISSFormer; (**e**) TransCeption; (**f**) D1CD2U-Net.

To evaluate the generalization capability of the models, the 3D-IRCADb01 dataset was employed as an independent external test set, enabling a comprehensive assessment of each model’s performance on previously unseen data. This section presents a comparison of the segmentation results on the 3D-IRCADb01 dataset, further demonstrating the superior performance of the proposed D1CD2U-Net model. Similarly to the internal test set, in Figure 16, panel (a) displays the original liver tumor image, (b) shows the ground truth annotation, while panels (c) through (f) present the segmentation results obtained by U-Net, FCN, LinkNet, and Unext, respectively. Figure 17 illustrates the segmentation results of AG-Net, DeeplabV3+, PSPNet, MISSFormer, and TransCeption in panels (a) through (e), with panel (f) showing the result produced by the proposed D1CD2U-Net model. The detailed segmentation results are presented in Figure 16 and Figure 17. The original images in Figure 16, from top to bottom, correspond to Example 4, Example 5, and Example 6, respectively.

In the case of small liver tumors shown in the first row and second column, all models except D1CD2U-Net and Unext failed to accurately identify the tumor regions, exhibiting severe under-segmentation. This highlights the inherent difficulty of achieving precise segmentation for small targets. In the second row, the superiority of D1CD2U-Net becomes even more evident, as it clearly distinguishes the tumor from surrounding tissues, whereas other models show varying degrees of insufficient detection. When dealing with multiple, non-contiguous tumor regions, as illustrated in the third row, D1CD2U-Net delivers particularly outstanding segmentation results. It not only avoids the common tumor merging issue (i.e., over-segmentation) observed in other models but also successfully identifies more tumor regions, demonstrating its high segmentation accuracy and capability. These results not only validate the effectiveness of D1CD2U-Net in complex scenarios but also reflect its superior capability in feature extraction and discrimination.

The remarkable performance of D1CD2U-Net on the external test set can be attributed to its unique architectural design. Firstly, the integration of the Deformable Large Kernel Attention mechanism enables the encoder to capture richer and more fine-grained image features, laying a solid foundation for accurate segmentation. Secondly, the inclusion of a Context Extraction mechanism significantly enhances the model’s ability to capture global information, helping it to understand local details while maintaining awareness of overall anatomical structures. Finally, the introduction of the Dual Cross Attention mechanism further promotes effective feature fusion and utilization, enabling more efficient and accurate integration of multi-scale and multi-level information. These three advantages work synergistically to equip D1CD2U-Net with strong competitiveness and broad application potential in the field of liver tumor segmentation.

### 6.7. Quantitative Analysis Based on Liver Tumor Examples

In liver tumor semantic segmentation, even models with strong overall performance may exhibit boundary blurring or fine-detail errors in specific scenarios. To comprehensively evaluate the applicability and limitations of the model, this study conducted a quantitative analysis of the visualized results. Tumor size was estimated based on pixel count, and segmentation performance metrics were calculated for each sample group to reveal performance variations under different conditions. Specifically, the Dice coefficient was used to assess edge accuracy, IoU measured segmentation region consistency, and the segmentation pixel error indicated whether the model exhibited over-segmentation (positive values) or under-segmentation (negative values). Detailed results are presented in Table 9, Table 10 and Table 11.

Overall, D1CD2U-Net demonstrates a clear advantage across different examples. It outperforms conventional convolutional networks and recent Transformer-based methods in both Dice and IoU metrics, with particularly notable improvements in Examples 1 and 4, highlighting its robustness in complex boundaries and low-contrast regions. Furthermore, analysis of pixel-wise segmentation errors shows that D1CD2U-Net achieves a more balanced trade-off between over-segmentation and under-segmentation, with overall lower error values compared to other methods, indicating higher precision in edge fitting and target coverage.

Comparison across different examples reveals some variability in model performance. In Examples 2 and 3, methods such as U-Net and AG-Net also achieved relatively high Dice scores, but they showed pronounced instability in other examples. In contrast, D1CD2U-Net maintained more consistent performance. Notably, in Example 4, conventional deep networks often yielded Dice and IoU values close to zero, reflecting difficulties in segmenting low-contrast or boundary-ambiguous regions, whereas D1CD2U-Net retained high accuracy, demonstrating strong generalization across samples. Nonetheless, Example 6 still exhibited boundary blurring and substantial negative errors, indicating limitations in small-volume lesions or low signal-to-noise scenarios.

Despite the overall superior performance, certain limitations remain. First, segmentation boundaries are still occasionally blurred or incomplete, particularly for small tumors or when the grayscale contrast between the tumor and liver parenchyma is low, indicating limited precision in localization and contour delineation. Second, group-wise error analysis shows that the model exhibits variable stability across lesions of different sizes and contrasts, suggesting a dependence on the underlying image feature distribution. Additionally, substantial negative errors in individual cases indicate potential omission of lesion regions, limiting applicability in clinical tasks that demand high sensitivity.

To address these limitations, future research can focus on several directions. First, incorporating uncertainty-based approaches could enable adaptive refinement of boundary-ambiguous regions, reducing errors in complex edges. Second, integrating cross-modal fusion strategies may enhance the model’s adaptability to lesions of varying size and contrast. Additionally, implementing group-wise evaluation and hard example mining during training could improve recognition of small or low-contrast lesions. Finally, combining interpretability analysis techniques could reveal the model’s attention mechanisms across different feature spaces, providing more reliable insights for clinical applications.

## 7. Conclusions

This study proposes a liver tumor segmentation approach that integrates multi-scale deformable feature fusion and global perception. The segmentation performance is significantly improved through three key innovations: (1) A Deformable Large Kernel Attention mechanism is introduced during feature extraction, effectively enhancing the encoder’s ability to capture multi-scale features. (2) A Dual Cross Attention mechanism is adopted to replace conventional skip connections, significantly improving the modeling of long-range dependencies and reducing semantic inconsistencies between encoder levels and between the encoder and decoder. (3) A Context Extraction module is designed to replace the standard bottleneck layer, thereby enhancing global context modeling and enabling more accurate localization of liver tumor regions. Experimental results demonstrate that the proposed method outperforms existing mainstream approaches in segmentation accuracy. Ablation studies further validate the effectiveness and synergy of each individual component. Moreover, evaluation on an independent test set confirms the strong generalization capability of the model. This study establishes a novel framework for liver tumor segmentation while offering insights for medical image segmentation module design.

## Figures and Tables

**Table 1:** Detailed dataset partitioning results.

Dataset	Internal Training Set	Internal Validation Set	Internal Test Set	External Test Set
LiTS	94	12	12	None
MSD Task08	243	30	30	None
3D-IRCADb01	None	None	None	15
Total	337	42	42	15

**Table 2:** Experimental environment and hyperparameter settings.

Category	Item	Configuration/Description
Hardware	Operating System	Ubuntu 22.04
	Processor	Intel Core i7-9700K (3.80 GHz)
	GPU	NVIDIA GeForce GTX 2080 Ti
	Memory	32 GB
Software	Framework	PyTorch 1.12.1
	Programming Language	Python 3.7.13
	GPU Acceleration Library	CUDA 11.6, cuDNN 8.5.x
Training Settings	Number of Epochs	50
	Batch Size	4
	Learning Rate Scheduler	Cosine Annealing
	Initial Learning Rate	10^−4^
	Minimum Learning Rate	10^−7^
	Optimizer	Adam

**Table 3:** Ablation study results.

	Precision	Recall	Dice	IoU	MIoU	Accuracy	MPA
U-Net	0.8478	0.6849	0.7579	0.6100	0.8021	0.9943	0.8417
CD2U-Net	0.8747	0.7300	0.7965	0.6609	0.8280	0.9951	0.8643
D1CU-Net	0.8687	0.7439	0.8016	0.6684	0.8319	0.9952	0.8712
D1D2U-Net	0.8755	0.6962	0.7756	0.6335	0.8141	0.9948	0.8474
D1CD2U-Net	0.8778	0.7570	0.8133	0.6848	0.8401	0.9955	0.8778

**Table 4:** Comparison results on the internal test set.

	Precision	Recall	Dice	IoU	MIoU	Accuracy	MPA
U-Net	0.8478	0.6849	0.7579	0.6100	0.8021	0.9943	0.8417
FCN	0.8258	0.6995	0.7574	0.6095	0.8018	0.9942	0.8488
DeeplabV3+	0.8562	0.6777	0.7566	0.6084	0.8014	0.9943	0.8381
PSPNet	0.8464	0.7007	0.7680	0.6217	0.808	0.9944	0.8495
Unext	0.7785	0.6858	0.7287	0.5739	0.7836	0.9934	0.8416
LinkNet	0.8698	0.6789	0.7632	0.6162	0.8053	0.9945	0.8388
AG-Net	0.8595	0.6999	0.7729	0.6281	0.8113	0.9946	0.8492
MISSFormer	0.7643	0.6375	0.6956	0.5328	0.7627	0.9927	0.8175
TransCeption	0.7307	0.5812	0.6482	0.4787	0.7352	0.9918	0.7892
D1CD2U-Net	0.8778	0.7570	0.8133	0.6848	0.8401	0.9955	0.8778

**Table 5:** Comparison results on the external test set.

	Precision	Recall	Dice	IoU	MIoU	Accuracy	MPA
U-Net	0.9402	0.6926	0.7983	0.6634	0.8300	0.9965	0.8461
FCN	0.8631	0.7074	0.7769	0.6360	0.8160	0.9960	0.8531
DeeplabV3+	0.9130	0.7216	0.8059	0.6752	0.8359	0.9966	0.8605
PSPNet	0.9032	0.7076	0.7937	0.6577	0.8270	0.9964	0.8534
Unext	0.8717	0.6980	0.7743	0.6329	0.8145	0.9960	0.8485
LinkNet	0.9201	0.7158	0.8049	0.6739	0.8352	0.9966	0.8576
AG-Net	0.9419	0.7068	0.8072	0.6773	0.8370	0.9967	0.8532
MISSFormer	0.9155	0.6867	0.7842	0.6458	0.8210	0.9963	0.8430
TransCeption	0.8885	0.6883	0.7756	0.6335	0.8148	0.9961	0.8437
D1CD2U-Net	0.9607	0.7421	0.8376	0.7203	0.8587	0.9972	0.8709

**Table 6:** Dice coefficient and IoU on internal test set.

	Dice	IoU
U-Net	0.7579 ± 0.0103 *	0.610 ± 0.0133 *
FCN	0.7574 ± 0.0124 *	0.6095 ± 0.0158 *
DeeplabV3+	0.7566 ± 0.0190 *	0.6084 ± 0.0241 *
PSPNet	0.7680 ± 0.0020 *	0.6217 ± 0.0040 *
Unext	0.7287 ± 0.0148 *	0.5739 ± 0.0185 *
LinkNet	0.7632 ± 0.0079 *	0.6162 ± 0.0111 *
AG-Net	0.7729± 0.0090 *	0.6281 ± 0.0107 *
MISSFormer	0.6956 ± 0.0140 *	0.5328 ± 0.0160 *
TransCeption	0.6482 ± 0.0039 *	0.4787 ± 0.0049 *
D1CD2U-Net	0.8133 ± 0.0012	0.6848 ± 0.0021

**Table 7:** Dice coefficient and IoU on the external test set.

	Dice	IoU
U-Net	0.7983 ± 0.0110 *	0.6634 ± 0.0160 *
FCN	0.7769 ± 0.0077 *	0.6360 ± 0.0098 *
DeeplabV3+	0.8059 ± 0.0025 *	0.6752 ± 0.0033 *
PSPNet	0.7937 ± 0.0080 *	0.6577 ± 0.0113 *
Unext	0.7743 ± 0.0038 *	0.6329 ± 0.0059 *
LinkNet	0.8049 ± 0.0022 *	0.6739 ± 0.0028 *
AG-Net	0.8072 ± 0.0040 *	0.6773 ± 0.0053 *
MISSFormer	0.7842 ± 0.0045 *	0.6458 ± 0.0063 *
TransCeption	0.7756 ± 0.0076 *	0.6335 ± 0.0102 *
D1CD2U-Net	0.8376 ± 0.0004	0.7203 ± 0.0006

**Table 8:** Detailed Results of model complexity and computational cost.

Model	Parameters	FLOPs	Training Time	Inference Time 1	Inference Time 2
U-Net	31.04 M	54.82 G	5 h 34 min	0.0261 s	0.0264 s
FCN	18.64 M	25.50 G	2 h 3 min	0.0177 s	0.0193 s
DeeplabV3+	5.81 M	6.61 G	9 h 22 min	0.0205 s	0.0229 s
PSPNet	2.41 M	2.51 G	1 h 53 min	0.0191 s	0.0211 s
Unext	0.25 M	0.10 G	1 h 46 min	0.0148 s	0.0159 s
LinkNet	11.53 M	3.04 G	1 h 45 min	0.0162 s	0.0176 s
AG-Net	9.34 M	16.57 G	10 h 1 min	0.0325 s	0.0335 s
MISSFormer	35.45 M	7.26 G	5 h 27 min	0.0339 s	0.0369 s
TransCeption	22.54 M	5.18 G	6 h 34 min	0.0325 s	0.0335 s
D1CD2U-Net	32.05 M	114.28 G	62 h 24 min	0.1032 s	0.1021 s

**Table 9:** Dice coefficients for individual examples.

	Example 1	Example 2	Example 3	Example 4	Example 5	Example 6
U-Net	0.2085	0.9363	0.9222	0.2212	0.7038	0.8777
FCN	0.1740	0.8031	0.8999	0.1442	0.6930	0.8600
LinkNet	0.1649	0.8467	0.8867	0	0.6692	0.8745
Unext	0.0801	0.7150	0.8034	0.7953	0.7765	0.7851
AG-Net	0.2024	0.9374	0.9085	0	0.7241	0.7865
DeeplabV3+	0.9033	0.8945	0.9023	0	0.7453	0.8464
PSPNet	0.2083	0.7630	0.8868	0	0.8108	0.8112
MISSFormer	0.0998	0.6013	0.8715	0	0.8227	0.8697
TransCeption	0.0935	0.8475	0.8298	0.1878	0.8210	0.8342
D1CD2U-Net	0.8708	0.8699	0.9183	0.8409	0.8840	0.8290

**Table 10:** IoU for individual examples.

	Example 1	Example 2	Example 3	Example 4	Example 5	Example 6
U-Net	0.1164	0.8803	0.8557	0.1244	0.5430	0.7820
FCN	0.0953	0.6710	0.8181	0.0777	0.5302	0.7543
LinkNet	0.0899	0.7342	0.7965	0	0.5028	0.7770
Unext	0.0417	0.5564	0.6714	0.6602	0.6347	0.6462
AG-Net	0.1126	0.8822	0.8324	0	0.5676	0.6481
DeeplabV3+	0.8236	0.8092	0.8220	0	0.5940	0.7336
PSPNet	0.1163	0.6168	0.7966	0	0.6817	0.6824
MISSFormer	0.0525	0.4299	0.7723	0	0.6989	0.7694
TransCeption	0.0490	0.7354	0.7092	0.1036	0.6963	0.7155
D1CD2U-Net	0.7712	0.7698	0.8489	0.7255	0.7921	0.7080

**Table 11:** Segmentation pixel error for individual examples.

	Example 1	Example 2	Example 3	Example 4	Example 5	Example 6
U-Net	+3184	+39	−149	−169	−967	−539
FCN	+3569	+38	−792	−178	−994	+358
LinkNet	+3620	+103	+50	−193	−1052	−49
Unext	+1425	+207	+1515	+44	−773	−563
AG-Net	+3681	+55	−971	−193	−915	−1314
DeeplabV3+	+5	+92	−1154	−193	−859	−848
PSPNet	+2805	+224	+1501	−193	−660	−136
MISSFormer	+3248	+561	−19	−193	−610	+19
TransCeption	+3522	+154	+223	−173	−495	−56
D1CD2U-Net	+109	+128	−11	−34	−404	−1325

## Data Availability

No new data were created or analyzed in this study. The original contributions presented in the study are included in the article, further inquiries can be directed to the corresponding authors.

## References

[1] Ying H., Liu X., Zhang M., Ren Y., Zhen S., Wang X., Liu B., Hu P., Duan L., Cai M. (2024). A multicenter clinical AI system study for detection and diagnosis of focal liver lesions. Nat. Commun..

[2] Chen J.G., Zhang Y.H., Lu J.H., Kensler T.W. (2024). Liver cancer etiology: Old issues and new perspectives. Curr. Oncol. Rep..

[3] Grover S., Gupta S. (2024). Automated diagnosis and classification of liver cancers using deep learning techniques: A systematic review. Discov. Appl. Sci..

[4] Baj J., Kołodziej M., Kobak J., Januszewski J., Syty K., Portincasa P., Forma A. (2024). Significance of Immune and Non-Immune Cell Stroma as a Microenvironment of Hepatocellular Carcinoma—From Inflammation to Hepatocellular Carcinoma Progression. Int. J. Mol. Sci..

[5] Hosseini S.V.S. (2024). Liver Cancer Surgery Technique. Eurasian J. Chem. Med. Pet. Res..

[6] Chen H., He Y., Jia W. (2020). Precise hepatectomy in the intelligent digital era. Int. J. Biol. Sci..

[7] Gundavda K.K., Patkar S., Varty G.P., Shah N., Velmurugan K., Goel M. (2024). Liver Resection for Hepatocellular Carcinoma: Recent Advances. J. Clin. Exp. Hepatol..

[8] Naaqvi Z., Haider M.A., Faheem M.R., Ain Q.U., Nawaz A., Ullah U. (2024). Modified U-Net Model for Segmentation and Classification of Liver Cancer Using Ct Images. J. Comput. Biomed. Inform..

[9] Wei T., Wang Y., Zhang Y., Wang Y., Zhao L. (2024). Boundary-sensitive segmentation of small liver lesions. IEEE J. Biomed. Health Inform..

[10] Meng K., Gong G., Liu R., Du S., Yin Y. (2024). Advances in gross tumor target volume determination in radiotherapy for patients with hepatocellular carcinoma. Front. Oncol..

[11] Jeong B., Choi S.J., Choi S.H., Jang H.J., Byun J.H., Won H.J., Shin Y.M. (2024). LI-RADS threshold growth based on tumor growth rate can improve the diagnosis of hepatocellular carcinoma ≤ 3.0 cm. Eur. Radiol..

[12] Wu C., Chen Q., Wang H., Guan Y., Mian Z., Huang C., Ruan C., Song Q., Jiang H., Pan J. (2024). A review of deep learning approaches for multimodal image segmentation of liver cancer. J. Appl. Clin. Med. Phys..

[13] Hu C., Xia T., Cui Y., Zou Q., Wang Y., Xiao W., Ju S., Li X. (2024). Trustworthy multi-phase liver tumor segmentation via evidence-based uncertainty. Eng. Appl. Artif. Intell..

[14] Mahdy L.N., Ezzat K.A., Torad M., Hassanien A.E. (2020). Automatic segmentation system for liver tumors based on the multilevel thresholding and electromagnetism optimization algorithm. Int. J. Imaging Syst. Technol..

[15] Revathy P., Sadasivam V. (2016). Analysis of Healthy versus Tumor Pixels Based on Segmentation Techniques for High Intensity Focused Ultrasound Interventions. J. Med. Imaging Health Inform..

[16] Baâzaoui A., Barhoumi W., Ahmed A., Zagrouba E. (2017). Semi-automated segmentation of single and multiple tumors in liver CT images using entropy-based fuzzy region growing. Irbm.

[17] Anter A.M., Hassenian A.E. (2019). CT liver tumor segmentation hybrid approach using neutrosophic sets, fast fuzzy c-means and adaptive watershed algorithm. Artif. Intell. Med..

[18] Roy S., Das D., Lal S., Kini J. (2023). Novel edge detection method for nuclei segmentation of liver cancer histopathology images. J. Ambient Intell. Humaniz. Comput..

[19] Smeets D., Loeckx D., Stijnen B., De Dobbelaer B., Vandermeulen D., Suetens P. (2010). Semi-automatic level set segmentation of liver tumors combining a spiral-scanning technique with supervised fuzzy pixel classification. Med. Image Anal..

[20] Xu L., Zhu Y., Zhang Y., Yang H. (2020). Liver segmentation based on region growing and level set active contour model with new signed pressure force function. Optik.

[21] Shelhamer E., Long J., Darrell T. (2017). Fully convolutional networks for semantic segmentation. IEEE Trans. Pattern Anal. Mach. Intell..

[22] Sun C., Guo S., Zhang H., Li J., Chen M., Ma S., Jin L., Liu X., Li X., Qian X. (2017). Automatic segmentation of liver tumors from multiphase contrast-enhanced CT images based on FCNs. Artif. Intell. Med..

[23] Christ P.F., Ettlinger F., Grün F., Elshaera M.E.A., Lipkova J., Schlecht S., Ahmaddy F., Tatavarty S., Bickel M., Bilic P. (2017). Automatic liver and tumor segmentation of CT and MRI volumes using cascaded fully convolutional neural networks. arXiv.

[24] Zheng S., Fang B., Li L., Gao M., Wang Y., Peng K. (2020). Automatic liver tumour segmentation in CT combining FCN and NMF-based deformable model. Comput. Methods Biomech. Biomed. Eng. Imaging Vis..

[25] Ronneberger O., Fischer P., Brox T. (2015). U-net: Convolutional networks for biomedical image segmentation. Medical Image Computing and Computer-Assisted Intervention–MICCAI 2015, Proceedings of the 18th International Conference, Munich, Germany, 5–9 October 2015.

[26] Sahli H., Ben Slama A., Labidi S. (2022). U-Net: A valuable encoder-decoder architecture for liver tumors segmentation in CT images. J. X-Ray Sci. Technol..

[27] Ayalew Y.A., Fante K.A., Mohammed M.A. (2021). Modified U-Net for liver cancer segmentation from computed tomography images with a new class balancing method. BMC Biomed. Eng..

[28] Seo H., Huang C., Bassenne M., Xiao R., Xing L. (2019). Modified U-Net (mU-Net) with incorporation of object-dependent high level features for improved liver and liver-tumor segmentation in CT images. IEEE Trans. Med. Imaging.

[29] Sabir M.W., Khan Z., Saad N.M., Khan D.M., Al-Khasawneh M.A., Perveen K., Qayyum A., Azhar Ali S.S. (2022). Segmentation of liver tumor in CT scan using ResU-Net. Appl. Sci..

[30] Li X., Chen H., Qi X., Dou Q., Fu C.W., Heng P.A. (2018). H-DenseUNet: Hybrid densely connected UNet for liver and tumor segmentation from CT volumes. IEEE Trans. Med. Imaging.

[31] Zhou Z., Rahman Siddiquee M.M., Tajbakhsh N., Liang J. (2018). Unet++: A nested u-net architecture for medical image segmentation. Deep Learning in Medical Image Analysis and Multimodal Learning for Clinical Decision Support, Proceedings of the 4th International Workshop, DLMIA 2018, and 8th International Workshop, ML-CDS 2018, Held in Conjunction with MICCAI 2018, Granada, Spain, 20 September 2018.

[32] Wang J., Peng Y., Jing S., Han L., Li T., Luo J. (2023). A deep-learning approach for segmentation of liver tumors in magnetic resonance imaging using UNet++. BMC Cancer.

[33] Peng Q., Yan Y., Qian L., Suo S., Guo Y., Xu J., Wang Y. (2022). Liver tumor segmentation and classification using FLAS-UNet++ and an improved DenseNet. Technol. Health Care.

[34] Li H., Liang B. (2023). Liver tumor computed tomography image segmentation based on an improved U-Net model. Appl. Sci..

[35] Li J., Liu K., Hu Y., Zhang H., Heidari A.A., Chen H., Zhang W., Algarni A.D., Elmannai H. (2023). Eres-UNet++: Liver CT image segmentation based on high-efficiency channel attention and Res-UNet++. Comput. Biol. Med..

[36] Kushnure D.T., Talbar S.N. (2022). HFRU-Net: High-level feature fusion and recalibration unet for automatic liver and tumor segmentation in CT images. Comput. Methods Programs Biomed..

[37] Zang L., Liang W., Ke H., Chen F., Shen C. (2023). Research on liver cancer segmentation method based on PCNN image processing and SE-ResUnet. Sci. Rep..

[38] Li W., Jia M., Yang C., Lin Z., Yu Y., Zhang W. (2023). SPA-UNet: A liver tumor segmentation network based on fused multi-scale features. Open Life Sci..

[39] Liu L., Wu K., Wang K., Han Z., Qiu J., Zhan Q., Wu T., Xu J., Zeng Z. (2024). SEU^2^-Net: Multi-scale U^2^-Net with SE attention mechanism for liver occupying lesion CT image segmentation. PeerJ Comput. Sci..

[40] Wang X., Zhang X., Wang G., Zhang Y., Shi X., Dai H., Liu M., Wang Z., Meng X. (2022). Transfusionnet: Semantic and spatial features fusion framework for liver tumor and vessel segmentation under jetsontx2. IEEE J. Biomed. Health Inform..

[41] Li X., Fang X., Yang G., Su S., Zhu L., Yu Z. (2023). Transu^2^-net: An effective medical image segmentation framework based on transformer and u^2^-net. IEEE J. Transl. Eng. Health Med..

[42] Zhang C., Lu J., Hua Q., Li C., Wang P. (2022). SAA-Net: U-shaped network with Scale-Axis-Attention for liver tumor segmentation. Biomed. Signal Process. Control..

[43] Jiang Y., Li Z., Chen X., Xie H., Cai J. (2024). Mlla-unet: Mamba-like linear attention in an efficient u-shape model for medical image segmentation. arXiv.

[44] Qamar S., Fazil M., Ahmad P., Muhammad G. (2025). SAMA-UNet: Enhancing Medical Image Segmentation with Self-Adaptive Mamba-Like Attention and Causal-Resonance Learning. arXiv.

[45] Zhu M., Cai Z., Fan Y., Chen Y. (2026). BPP-net: Bio-inspired parallel pathway network for liver and tumor segmentation. Biomed. Signal Process. Control..

[46] Lou M., Zhang S., Zhou H.Y., Yang S., Wu C., Yu Y. (2025). TransXNet: Learning both global and local dynamics with a dual dynamic token mixer for visual recognition. IEEE Trans. Neural Netw. Learn. Syst..

[47] Lou M., Yu Y. OverLoCK: An Overview-first-Look-Closely-next ConvNet with Context-Mixing Dynamic Kernels. Proceedings of the Computer Vision and Pattern Recognition Conference.

[48] Lou M., Fu Y., Yu Y. Sparx: A sparse cross-layer connection mechanism for hierarchical vision mamba and transformer networks. Proceedings of the AAAI Conference on Artificial Intelligence.

[49] Fu Y., Lou M., Yu Y. SegMAN: Omni-scale context modeling with state space models and local attention for semantic segmentation. Proceedings of the Computer Vision and Pattern Recognition Conference.

[50] Huang G., Zhu J., Li J., Wang Z., Cheng L., Liu L., Li H., Zhou J. (2020). Channel-attention U-Net: Channel attention mechanism for semantic segmentation of esophagus and esophageal cancer. IEEE Access.

[51] Zhu X., Cheng D., Zhang Z., Lin S., Dai J. An empirical study of spatial attention mechanisms in deep networks. Proceedings of the IEEE/CVF International Conference on Computer Vision 2019.

[52] Zhao H., Jia J., Koltun V. Exploring self-attention for image recognition. Proceedings of the IEEE/CVF Conference on Computer Vision and Pattern Recognition 2020.

[53] Azad R., Niggemeier L., Hüttemann M., Kazerouni A., Aghdam E.K., Velichko Y., Bagci U., Merhof D. Beyond self-attention: Deformable Large Kernel Attention for medical image segmentation. Proceedings of the IEEE/CVF Winter Conference on Applications of Computer Vision 2024.

[54] Wang H., Cao P., Wang J., Zaiane O.R. Uctransnet: Rethinking the skip connections in u-net from a channel-wise perspective with transformer. Proceedings of the AAAI Conference on Artificial Intelligence.

[55] Wang H., Cao P., Yang J., Zaiane O. (2024). Narrowing the semantic gaps in u-net with learnable skip connections: The case of medical image segmentation. Neural Netw..

[56] Ates G.C., Mohan P., Celik E. (2023). Dual Cross Attention for medical image segmentation. Eng. Appl. Artif. Intell..

[57] Gu Z., Cheng J., Fu H., Zhou K., Hao H., Zhao Y., Zhang T., Gao S., Liu J. (2019). Ce-net: Context encoder network for 2d medical image segmentation. IEEE Trans. Med. Imaging.

